# Binary architecture of the Na_v_1.2-β2 signaling complex

**DOI:** 10.7554/eLife.10960

**Published:** 2016-02-19

**Authors:** Samir Das, John Gilchrist, Frank Bosmans, Filip Van Petegem

**Affiliations:** 1 Department of Biochemistry and Molecular Biology, Life Sciences Institute, University of British Columbia, Vancouver, Canada; 2 Department of Physiology, Johns Hopkins University School of Medicine, Baltimore, United States; 3 Solomon H Snyder Department of Neuroscience, Johns Hopkins University School of Medicine, Baltimore, United States; Howard Hughes Medical Institute, Boston Children's Hospital, United States

**Keywords:** voltage-gated sodium channel, beta2 subunit, scn2b, spider toxin, X-ray structure, disulfide, <i>E. coli</i>, *Xenopus*

## Abstract

To investigate the mechanisms by which β-subunits influence Na_v_ channel function, we solved the crystal structure of the β2 extracellular domain at 1.35Å. We combined these data with known bacterial Na_v_ channel structural insights and novel functional studies to determine the interactions of specific residues in β2 with Na_v_1.2. We identified a flexible loop formed by ^72^Cys and ^75^Cys, a unique feature among the four β-subunit isoforms. Moreover, we found that ^55^Cys helps to determine the influence of β2 on Na_v_1.2 toxin susceptibility. Further mutagenesis combined with the use of spider toxins reveals that ^55^Cys forms a disulfide bond with ^910^Cys in the Na_v_1.2 domain II pore loop, thereby suggesting a 1:1 stoichiometry. Our results also provide clues as to which disulfide bonds are formed between adjacent Na_v_1.2 ^912/918^Cys residues. The concepts emerging from this work will help to form a model reflecting the β-subunit location in a Na_v_ channel complex.

**DOI:** http://dx.doi.org/10.7554/eLife.10960.001

## eLife digest

Our bodies run on electricity. The brain, heart and some other organs depend on small electrical signals that are generated by ions moving through specialized protein complexes that sit in the membrane surrounding a cell. One of these channels is a ‘sodium channel’, through which positively charged sodium ions move. Tiny changes in the structure of the sodium channel can cause severe conditions such as epilepsy and heart arrhythmias, so it is crucial that we know how it works

Sodium channels consist of different protein building blocks (called α and β) and it was not known exactly how these come together to form the full channel complex. However, previous studies hinted at which parts of the β building block make contact with the α protein.

Now, Das, Gilchrist et al. have been able to visualize the three-dimensional structure of the β building block of the sodium channel in extremely high detail by using a technique called X-ray crystallography. The level of detail in the structure also allowed the amino acids that make up the β building block to be identified.

Das, Gilchrist et al. then altered some of the amino acids in the sodium channel, and treated frog cells containing the mutant channel with a spider toxin that binds between the α and β building blocks. This revealed the location and identity of the exact contact points between the proteins. In the future, a full three-dimensional structure showing the α and β subunits bound together would yield invaluable information on how they cooperate to form the sodium channel complex and give insights into mutations that cause cardiac arrhythmias and epilepsy.

**DOI:** http://dx.doi.org/10.7554/eLife.10960.002

## Introduction

Voltage-gated sodium (Na_v_) channels are part of membrane-embedded signaling complexes that initiate the rising phase of action potentials, a crucial event in generating and propagating electrical signals throughout the human body (Hille, 2001; Catterall, 2012). As key components of these protein assemblies, β-subunits (1) modify Na_v_ channel-gating properties; (2) regulate channel trafficking and localization to distinct surface compartments; and (3) influence channel oligomerization (Calhoun and Isom, 2014; Namadurai et al., 2015). Moreover, β-subunits can alter the toxin pharmacology of Na_v_ channels (Gilchrist et al., 2014), a concept that has been exploited to detect their presence in heterologous expression systems or native tissues (Wilson et al., 2011; Zhang et al., 2013; Wilson et al., 2015; Gilchrist et al., 2013). Structurally, β-subunits are single-transmembrane segment glycoproteins with a short cytoplasmic C-terminal tail and a large V-type immunoglobulin (Ig) extracellular domain that may participate in homophilic and heterophilic interactions, cell adhesion, and cell migration (Calhoun and Isom, 2014; Namadurai et al., 2015; Brackenbury et al., 2008). Although all β-subunits belong to the Ig family, recent atomic resolution information for the β3 and β4 extracellular domain revealed substantial differences in their 3D structure (Gilchrist et al., 2013; Namadurai et al., 2014). Given their distinct features and functional roles, it has now become clear that each β-subunit structure should be obtained and assessed separately. Of the four known β-subunits and their splice variants (β1–4; gene names *Scn1b*-*Scn4b*) (Isom et al., 1992; Isom et al., 1995a; Morgan et al., 2000; Patino, 2011; Yu, 2003), β2 and β4 form a disulfide bond with an unidentified Cys within particular Na_v_ channel isoforms. In contrast, non-covalent interactions underlie β1 and β3 association with Na_v_ channels as well as other members of the voltage-gated ion channel family (Calhoun and Isom, 2014; Namadurai et al., 2015; Marionneau, 2012; Nguyen et al., 2012; Deschenes et al., 2008). Aberrant behavior of the ubiquitously expressed β2 and β4 subunits has been linked to disorders such as long-QT syndrome, atrial fibrillation, sudden infant death syndrome, and epilepsy, possibly through dysregulation of the Na_v_ channel signaling complex (Li et al., 2013; Medeiros-Domingo et al., 2007; Tan et al., 2010; Baum et al., 2014; Watanabe et al., 2009). Moreover, β2 has been implicated in neurodegenerative disorders and neuropathic pain and is therefore of potential interest for developing novel therapeutic strategies (O'Malley et al., 2009; Lopez-Santiago, 2006). Finally, β2 is targeted by secretase enzymes, an observation that suggests a potential contribution to Alzheimer's disease (Gersbacher et al., 2010; Kim et al., 2005).

Despite accruing evidence supporting their clinical relevance, fundamental questions on the causal relationship between β2 mutations and disorders remain unanswered. In contrast, auxiliary proteins are the topic of herculean research efforts in other fields where their role as vital contributors to cellular function or in forming drug receptor sites is well established (Copits and Swanson, 2012; Gee et al., 1996; Milstein and Nicoll, 2008; Dolphin, 2012). To begin to appreciate β2 function and lay the foundations for constructing an interacting model with Na_v_ channels, we first need to define the mechanism by which β2 regulates Na_v_ channel function. In particular, knowledge of the relative orientation of both partners will help to assess whether a mutation modifies channel function or influences complex assembly. To this end, we solved the crystal structure of the extracellular β2 domain at 1.35Å and found a ^55^Cys-containing binding region that modulates Na_v_1.2 toxin susceptibility by β2. Next, we exploited a combination of Na_v_ channel mutagenesis, biochemistry, and β2-induced alterations in spider toxin pharmacology to uncover a disulfide bond between ^55^Cys and a partnering Cys located in the domain II S5-S6 pore loop of Na_v_1.2. Remarkably, Na_v_1.5 and close relatives Na_v_1.8/Na_v_1.9 do not possess a corresponding Cys, which may explain a lack of β2 effect on Na_v_1.5 toxin pharmacology. In concert with the available structural information on bacterial Na_v_ channels (Payandeh et al., 2012; 2011; Shaya et al., 2014; Zhang et al., 2012), our results provide conceptual insights into the location of the β2-subunit in the Na_v_ channel-signaling complex.

## Results

### Crystal structure of the human β2 extracellular region

**Figure 1.**
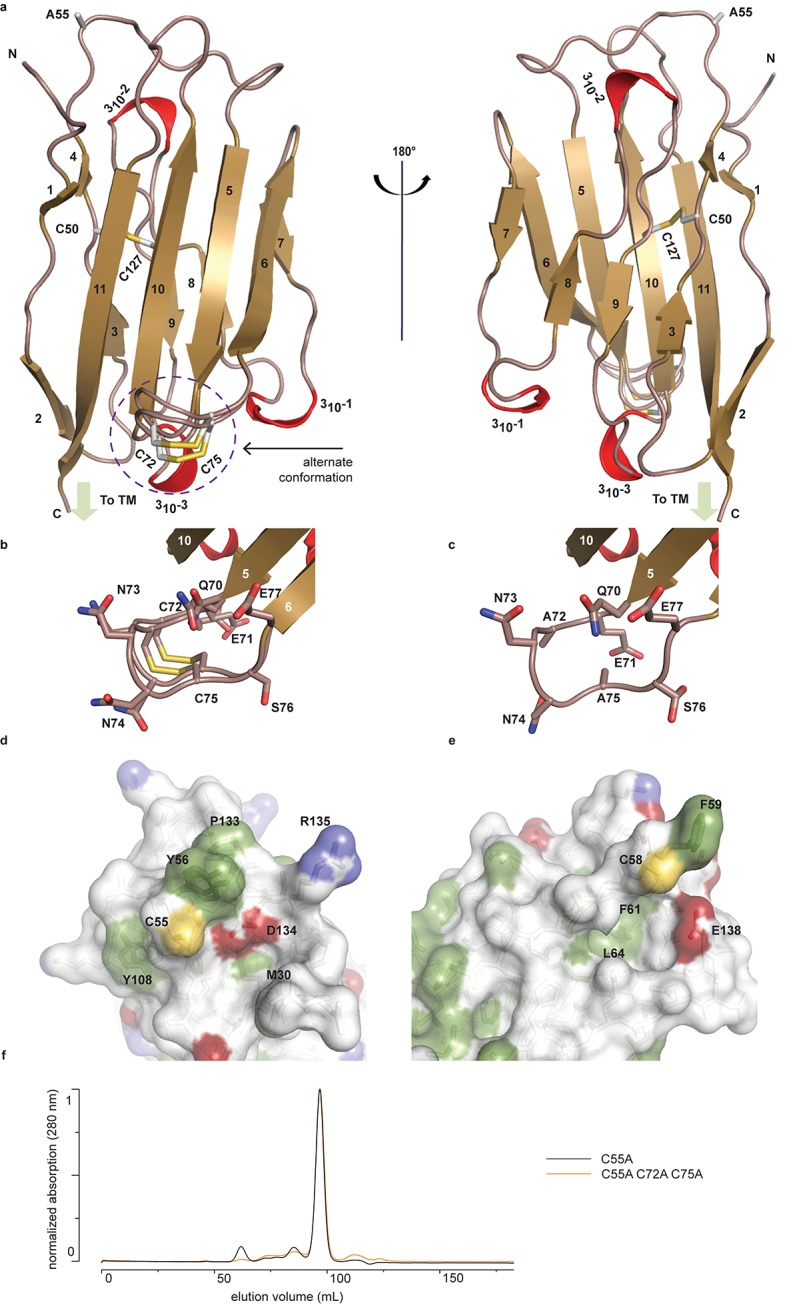
Crystal structure of hβ2. (**a**) Cartoon representation of the hβ2 (C55A) extracellular domain crystal structure, showing β strands in gold and 3_10_ helices in red. Cysteine side chains, as well as the ^55^Ala residue, are shown in stick representation. Positions of N-terminus (N) and C-terminus (C) are indicated. The loop containing the ^72^Cys- ^75^Cys disulfide bond is modeled in a dual conformation. (**b**) Detail of the dual conformation of the loop, showing all side chains in stick conformation. (**c**) Detail of the same loop in the C72A/C75A (C55A) mutant, shown from the same viewpoint as in panel (**b**). (**d,e**) Comparison of the surfaces of hβ2 (**d**) and hβ4 (**e**) surrounding the reactive cysteines (^55^Cys and ^58^Cys, respectively). Side chains of hydrophobic residues are shown in green, negatively charged carboxyl groups in red, and positively charged amino and guanidinium groups in blue. The position of the cysteine (which has been mutated to alanine to allow crystallization) is shown in yellow. (**f**) Size exclusion chromatograms (Preparative Superdex200) for hβ2 C55A and hβ2 C55/72/75A, which both elute as monomeric species. **DOI:** http://dx.doi.org/10.7554/eLife.10960.003 10.7554/eLife.10960.004Figure 1—source data 1.X-ray data collection and refinement statistics.**DOI:** http://dx.doi.org/10.7554/eLife.10960.004

**Figure 2.**
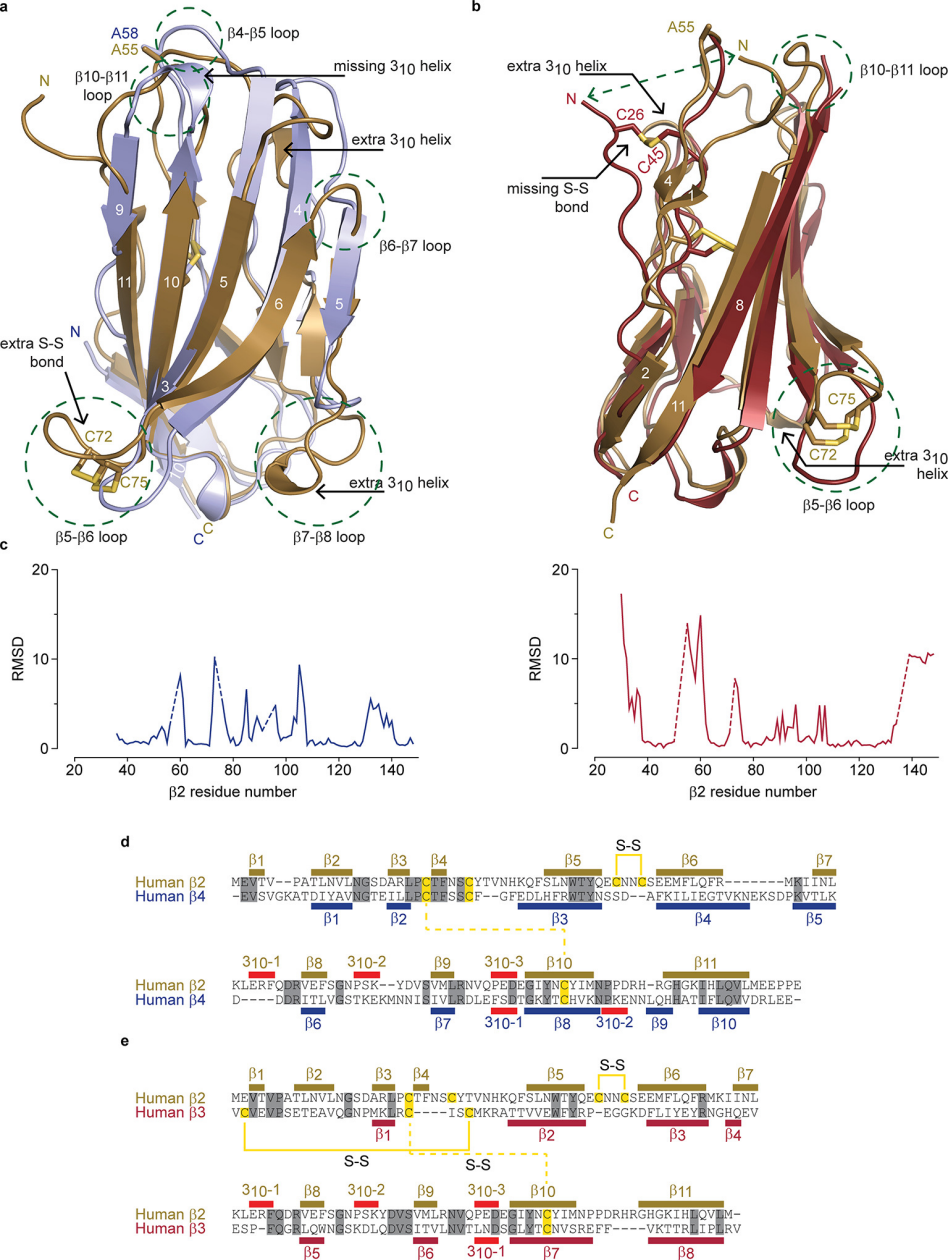
Structural comparison of hβ2 to hβ3 and hβ4. (**a**) Superposition of the crystal structures of hβ2 C55A (gold) and hβ4 C58A (blue) in cartoon representation. Cysteines or their equivalent residues (A55 and A58, respectively) are shown in sticks. The major differences are highlighted in the figure. (**b**) Superposition of crystal structures of hβ2 C55A (gold) and hβ3 (red). (**c**) Root-mean-square-deviation (RMSD) plots showing the RMSD values per residue for hβ4 C58A (left, blue plot) and hβ3 (right, red plot) relative to hβ2 after a superposition. The residue numbers below correspond to the hβ2 numbers. Sections for which there are no corresponding residues at indicated with dotted lines. (**d,e**) Shown are sequence alignments of the extracellular domains of (**d**) hβ2 versus hβ4 and (**e**) hβ2 versus hβ3. Conserved residues are highlighted in grey, and cysteines in yellow. Observed disulfide bonds are also labeled (S-S). Secondary structure elements found in the corresponding crystal structures are also shown. **DOI:** http://dx.doi.org/10.7554/eLife.10960.005

To begin to understand the functional relationship between human (h)β2 and hNa_v_ channels at an atomic level, we solved the crystal structure of the extracellular hβ2 domain at a resolution of 1.35Å (Figure 1a, Figure 1—source data 1). The construct encompasses residues 30–153 (Figure 2), and contains a single Cys mutation (C55A) to facilitate crystallization [The C55A mutation is located on the protein surface and therefore unlikely to affect the overall structure (Figure 1a,d).]. The hβ2 configuration displays an Ig-like fold, consisting of eleven β-strands and three 3_10_ helices. Other than the mutated Cys (C55A), this domain contains four additional cysteines that are arranged in two bonds. The first, intra-subunit bond, is strictly conserved among all four β-subunit isoforms and is mediated by ^50^Cys and ^127^Cys buried within the core where it links two opposing faces of the protein. The second intra-subunit bond is located within a loop that spans residues 70–77 and connects strand β_5_ to β_6_ via ^72^Cys - ^75^Cys (Figure 1a–b). This loop constitutes a unique feature of hβ2 since corresponding cysteines are absent in β1, β3, and β4. Remarkably, this region displays a dual conformation in the crystal structure which indicates a high degree of flexibility (Figure 1b).

**Figure 2—figure supplement 1.**
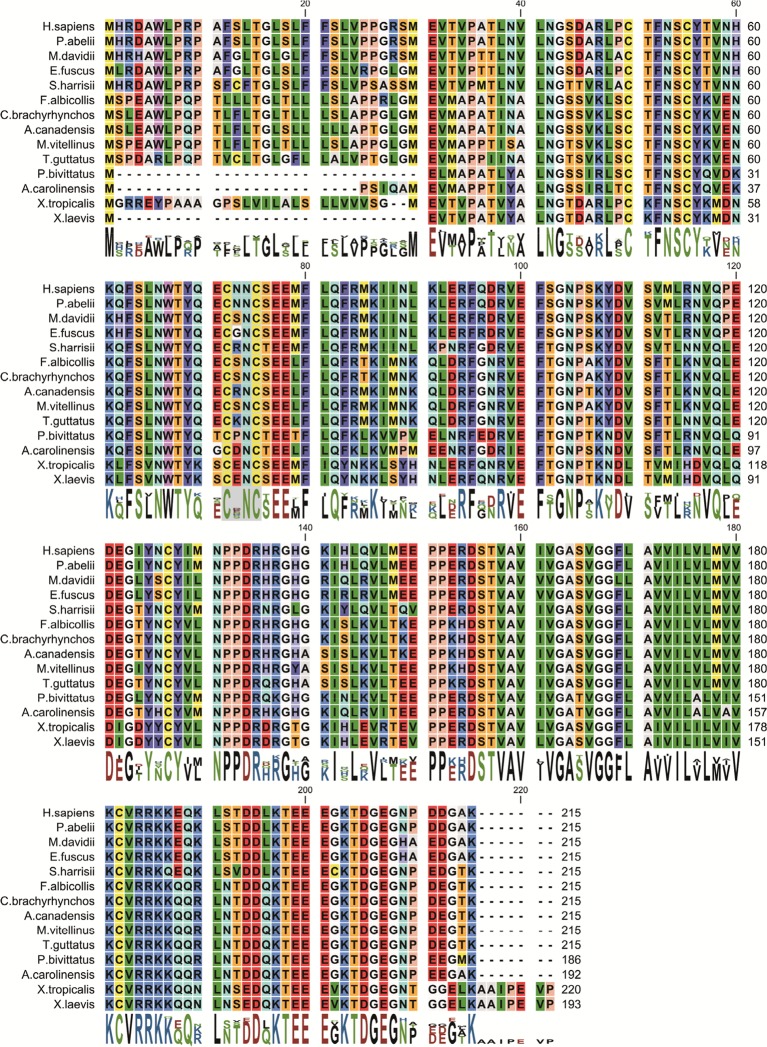
Amino acid sequence alignment of the β2 protein found in various organisms. Conserved cysteines ^72^Cys and ^75^Cys in hβ2 are indicated with a grey background. Residue numbers in hβ2 above sequences are shown as a reference. Protein accession numbers for sequences used are: *H. sapiens* (NP_004579.1), *P. abelii* (XP_002822587.1), *M. davidii* (XP_006761624.1), *E. fuscus* (XP_008148335.1), *S. harrisii* (XP_003764204.1), *T. guttatus* (XP_010214560.1), *C. brachyrhynchos* (XP_008635161.1), *F. albicollis* (XP_005058672.1), *A. canadensis* (XP_011600162.1), *M. vitellinus* (XP_008927155.1), *A. carolinensis* (XP_003229016.2), *P. bivittatus* (XP_007424304.1), *X. tropicalis* (NP_001116903.2), *X. laevis* (NP_001088105.2). **DOI:** http://dx.doi.org/10.7554/eLife.10960.006

Although the overall hβ2 extracellular domain exhibits a similar fold compared to that of the previously reported hβ3^11^ and hβ4^9^ structures, considerable differences are observed. Aside from the distinct ^72^Cys - ^75^Cys disulfide bond, there are extensive variances in loop lengths and β strands, indicating that hβ2 and hβ4 have diverged substantially (Figure 2a,c). Yet, at a protein sequence level, hβ2 is most closely related to hβ4 (26% identical; 48% conserved) (Figure 2d,e). Of note is that the N-terminal region of hβ2 is structured rather than disordered in hβ4, adding an additional short β strand (β_1_) which interacts with the novel β_4_ strand. The strand following the unique disulfide bond is shortened in hβ2 and two additional 3_10_ helices can be seen, whereas one that is present in hβ4, no longer exists in hβ2. A comparison with the hβ3 structure also shows substantial divergence in loop lengths at particular locations (Figure 2b,c). The hβ3 extracellular domain misses the additional N-terminal β-strand; instead, its N-terminus is anchored via a disulfide bridge between ^26^Cys and ^48^Cys, resulting in positional shifts in excess of 15Å.

We previously found that ^58^Cys on the surface of β4 is crucial in modulating Na_v_1.2 susceptibility to toxins from spider and scorpion venom (Gilchrist et al., 2013). The hβ2 subunit possesses a corresponding Cys at position 55 that is located in a longer loop which may result in an altered spatial position of this residue when compared to hβ4 (Figure 2a,c, Figure 1d,e). In addition, the residues immediately N-terminal to ^55^Cys are stabilized through a β-strand interaction between strands β_1_ and β_4_, both of which are absent in hβ4. Taken together, these observations suggest that the environment of this key Cys may differ in both isoforms. To further examine the coupling of hβ2 to hNa_v_1.2, we next determined the functional role of ^55^Cys as well as that of ^72^Cys and ^75^Cys.

### Mutating ^55^Cys in hβ2 restores hNa_v_1.2 toxin susceptibility

**Figure 3.**
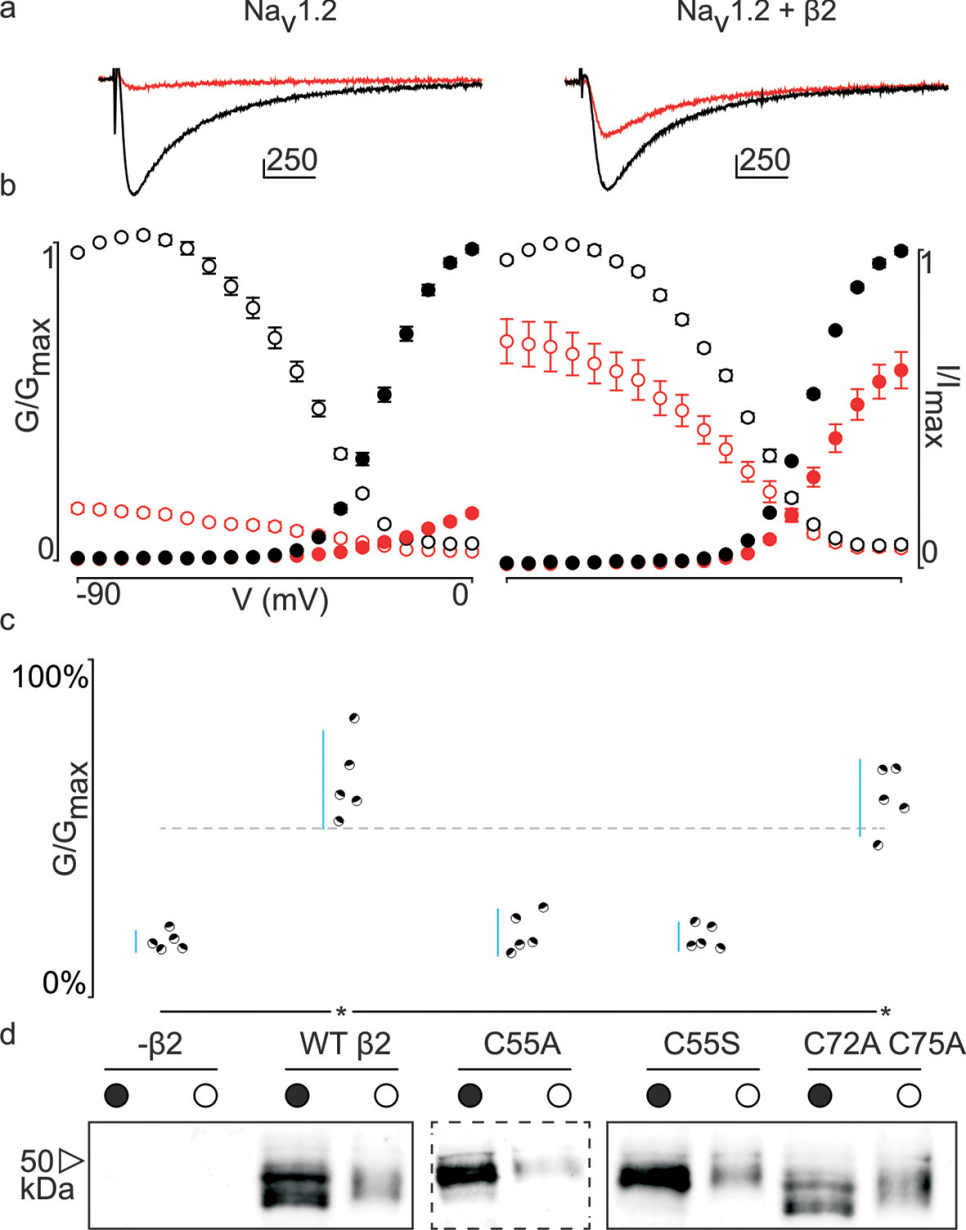
Effect of hβ2 on hNa_v_1.2 toxin pharmacology. (**a**) Co-expression of hNa_v_1.2 with hβ2 decreases the degree of inhibition by 100 nM ProTx-II. Left trace shows ProTx-II strongly inhibiting WT hNa_v_1.2 whereas right trace displays attenuated inhibition in the presence of hβ2. Black trace is control condition without toxin, red is in the presence of ProTx-II. Traces depict a 50 ms depolarization to -15 mV from -90 mV. Scale bar is 10 ms on horizontal axis and given nA vertically. (**b**) Normalized conductance-voltage (G-V, filled circles) and steady-state inactivation (I-V, open circles) relationships for hNa_v_1.2 with and without hβ2. Pre-toxin values are shown in black and post-toxin in red. Fit values can be found in Figure 3—source data 1. (**c**) Dot plot comparing hβ2 mutations by ability to prevent ProTx-II inhibition of hNa_v_1.2. Black circles represent individual oocytes; vertical axis shows percent of inhibition by ProTx-II at peak conductance. Blue lines represent a 95% confidence interval. hβ2 mutations are presented underneath the horizontal axis and label the lanes below in (**d**). Statistical significance (p<0.01) is indicated by an asterisk. (**d**) Western blot against the C-terminal myc-tag of hβ2. No signal is seen in the negative control but is observed for the WT hβ2 and all mutants, both in whole cell (filled circle) and surface (open circle) fractions. **DOI:** http://dx.doi.org/10.7554/eLife.10960.007 10.7554/eLife.10960.008Figure 3—source data 1.Table providing values for fits of the data presented in Figure 3 and Figure 3—figure supplement 2.G-V and SSI relationship data were fitted by a Boltzmann curve. V_1/2_ provides the midpoint voltage of the calculated curve (in mV) and Vc the unit-less slope, with standard error of the mean (SEM). Right column shows peak conductance after toxin treatment as a fraction of untreated peak conductance with the upper and lower bounds of the 95% confidence interval in parentheses, reflecting the data displayed in the dot plots.**DOI:** http://dx.doi.org/10.7554/eLife.10960.008

Previously, we and others discovered that β-subunits can manipulate the pharmacological sensitivities of Na_v_ channels (Wilson et al., 2011; Gilchrist et al., 2013; Doeser et al., 2014). In particular, β4 is capable of dramatically altering animal toxin binding to rat (r)Na_v_1.2a (Gilchrist et al., 2013). For example, the spider toxin ProTx-II (Middleton et al., 2002) binds to voltage-sensing domains (VSDs) I, II, and IV in rNa_v_1.2a (Bosmans et al., 2008), and is ∼five-fold less potent when β4 is present. [An intriguing concept is that ProTx-II may also bind directly to β4 (Gee et al., 1996); however, our isothermal calorimetry experiments do not support this notion (Figure 3—figure supplement 1).] To examine whether this protective ability extends to hβ2, we expressed hNa_v_1.2 in *Xenopus* oocytes and measured ProTx-II susceptibility without or in the presence of the β-subunit (Figure 3—source data 1, Figure 3—figure supplement 2). Similar to β4, we observe that the hβ2 subunit expresses abundantly and traffics to the membrane (Figure 3d) where it is able to reduce the degree of hNa_v_1.2 current inhibition by ProTx-II. Specifically, 100 nM ProTx-II reduces hNa_v_1.2 conductance to ∼17% of peak whereas the current remaining in the presence of hβ2 is typically more than ∼64% of peak conductance, thereby demonstrating a protective effect. Other gating parameters such as conductance-voltage (G–V) and channel availability relationships are unaffected (Figure 3—source data 1). Next, we sought to determine if ^55^Cys in hβ2 is involved in reducing hNa_v_1.2 susceptibility to ProTx-II by mutating this residue to an Ala. Indeed, the C55A mutant traffics to the membrane and causes ProTx-II inhibition of hNa_v_1.2 to resemble that of the wild-type (WT) channel without hβ2 present (Figure 3c,d, Figure 3—figure supplement 2). Although Ala wields a small side chain and is often employed in mutagenesis studies, we also replaced ^55^Cys with a Ser, as it most closely resembles Cys in terms of size and electric properties. Similar to WT hβ2 and C55A, the C55S mutation impaired neither channel expression nor surface trafficking (Figure 3d, Figure 3—figure supplement 2). Moreover, the extent of ProTx-II-induced hNa_v_1.2 inhibition in the presence of C55S is indistinguishable from that of the channel alone (Figure 3c, Figure 3—source data 1). Altogether, these results support the notion that hβ2 conveys ProTx-II protection to hNa_v_1.2 via ^55^Cys and may relate to previous work in which the loss of the covalent link between rβ2 and hNa_v_1.1 disrupts the targeting of rβ2 to nodes of Ranvier and to the axon initial segment in hippocampal neurons (Chen et al., 2012).

**Figure 3—figure supplement 1.**
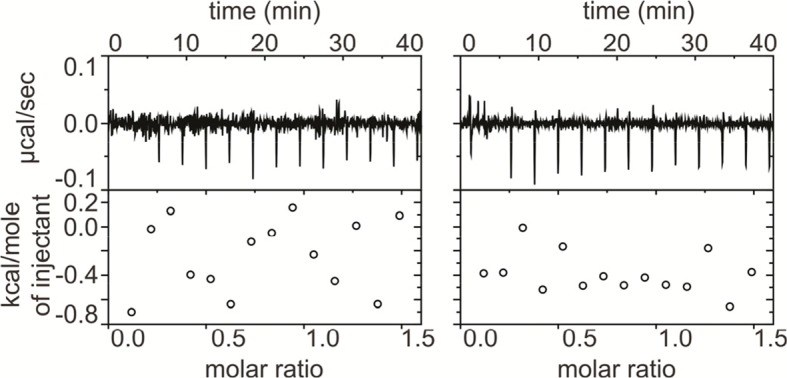
ProTx-II does not bind directly to β4. Isothermal calorimetry (ITC) experiments showing titration of 400 μM WT hβ4 extracellular domain into 40 μM ProTx-II (left) and into buffer (right). No significant heat differences are detected. **DOI:** http://dx.doi.org/10.7554/eLife.10960.009

**Figure 3—figure supplement 2.**
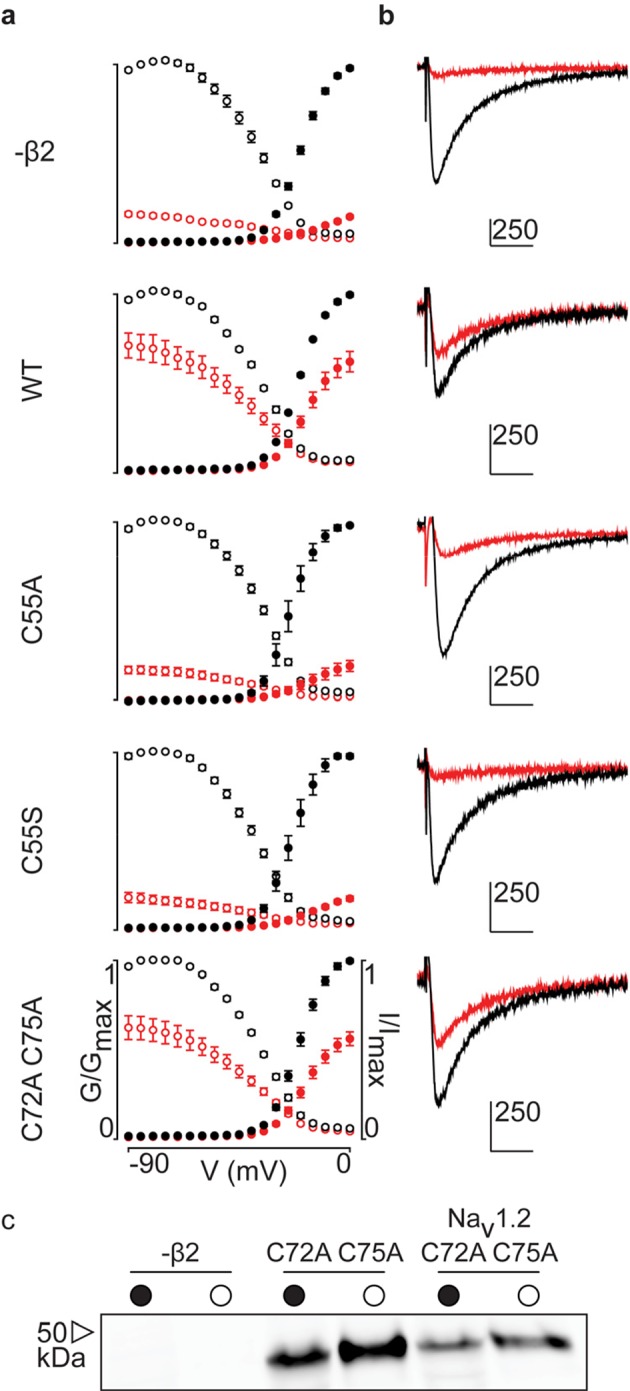
G-V and SSI relationships for hβ2 mutants. (**a**) Conductance-voltage (G-V, filled circles) and steady-state inactivation (SSI, open circles) relationships before 10nM ProTx-II addition are indicated in black and red in the presence of toxin. (**b**) Representative traces at -15 mV (holding potential of -90 mV) for the corresponding graphs seen in (**a**). Scale bar is 10 ms along horizontal axis and displayed nA along the vertical axis. (**c**) Western blot showing that the C72A C75A hβ2 mutant is expressed in whole cell (filled circle) and surface (open circle) fractions either alone (middle column) or with hNa_v_1.2 (right column). The hβ2 subunit was detected using an antibody against a C-terminal myc-tag. **DOI:** http://dx.doi.org/10.7554/eLife.10960.010

In addition to ^55^Cys, the hβ2 crystal structure reveals a unique motif bearing a protruding disulfide-stabilized loop formed by ^72^Cys and ^75^Cys (Figure 1a–b). Remarkably, this additional loop is highly conserved in almost all species that express β2, suggesting an evolutionary conserved contribution to function (see Figure 2, Figure 2—figure supplement 1). To determine if this loop regulates the gating or pharmacological influence of hβ2 on hNa_v_1.2, we mutated ^72^Cys and ^75^Cys to Ala (C72A C75A) but found that it is functionally indistinguishable from WT hβ2 (Figure 3c, Figure 3—figure supplement 2). The C72A C75A mutant localizes to the oocyte membrane surface without or with hNa_v_1.2 co-expression. Moreover, typical gating parameters and hNa_v_1.2 inhibition by 100 nM ProTx-II in the presence of C72A C75A is similar to that observed for the channel when co-expressed with WT hβ2 (Figure 3—source data 1). The lack of effect of the C72A C75A mutant on hNa_v_1.2 function suggests that this disulfide bond is not essential for folding, and that its disruption may not significantly affect the position or environment of ^55^Cys. To verify this hypothesis, we produced recombinant hβ2 extracellular domain containing three Cys mutations: C55A, C72A, and C75A. Size exclusion chromatography demonstrates that the mutant produces monomeric protein, indicating that the bond between ^72^Cys and ^75^Cys is unessential for folding (Figure 1f). Furthermore, we obtained a crystal structure of the triple mutant at 1.85Å which overlays well onto the C55A structure (Figure 1c). The only significant difference is situated in the loop containing both cysteines, which now displays a single conformation. Although the spatial organization of this loop does not seem to impact the ability of hβ2 to modulate hNa_v_1.2 gating or sensitivity to ProTx-II, this region may yet play a functional role in modulating other Na_v_ channel isoforms.

### The S5-S6 loop in domain II of hNa_v_1.2 contains an anchoring point for hβ2

**Figure 4.**
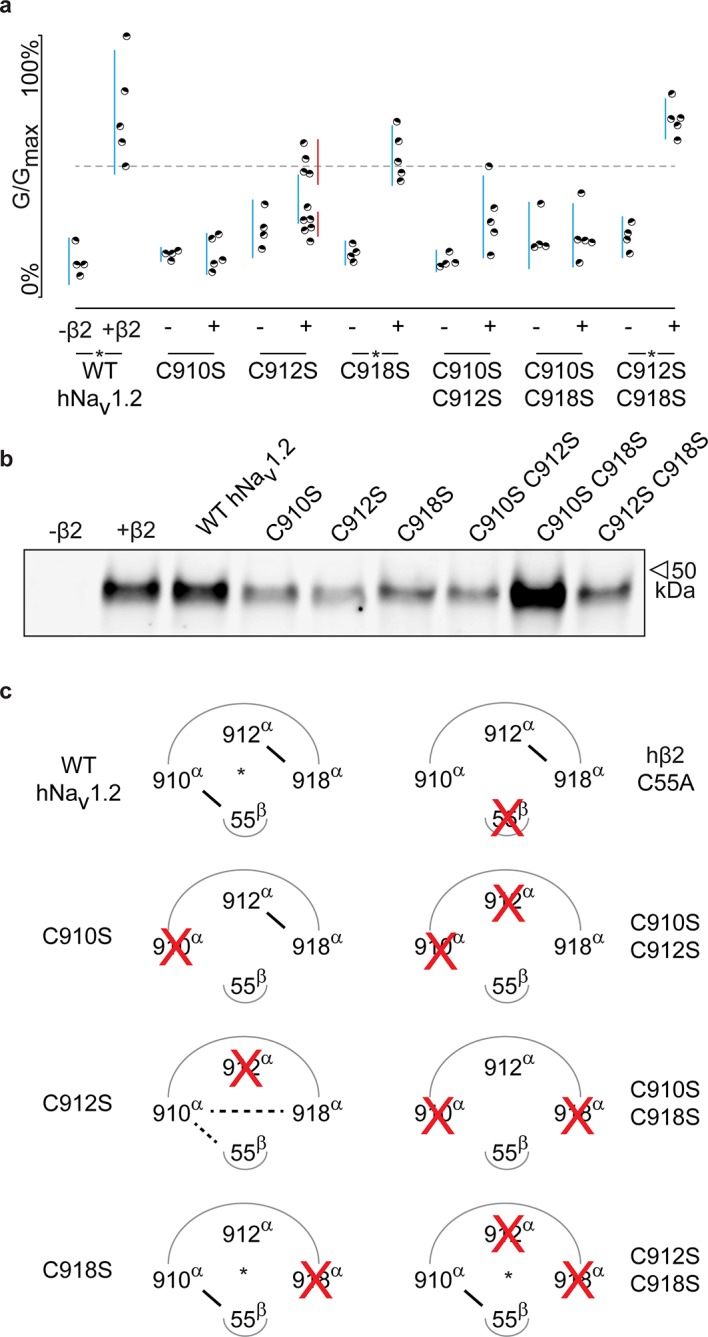
hβ2 forms a disulfide bond with ^910^Cys in hNa_v_1.2. (**a**) Dot plot showing degree of hNav1.2 mutant inhibition by 100 nM ProTx-II, with and without hβ2. Black circles represent single oocytes expressing the indicated constructs and currents were measured at peak conductance. Blue bars indicate 95% confidence interval, and the red bars are 95% confidence intervals for both populations in the C912S mutant co-expressed with hβ2. Statistical significance with p.01 is shown by an asterisk. (**b**) Western blot against a myc-tag reveals the presence of hβ2 in whole cell fraction of oocytes co-injected with hβ2 mRNA. (**c**) Schematic depiction of the proposed disulfide arrangement in hNa_v_1.2 mutants with hβ2. hNa_v_1.2 cysteines are displayed on the top arc with α (pore-forming subunit), while ^55^Cys of hβ2 is on the bottom arc with β. Black lines represent putative disulfide bonds and dashed lines indicate alternate possibilities. Red X symbolizes a Cys to Ser mutation and an asterisk denotes mutants protected against 100 nM ProTx-II. **DOI:** http://dx.doi.org/10.7554/eLife.10960.011 10.7554/eLife.10960.012Figure 4—source data 1.Table providing values for fits of the data presented in Figure 4 and Figure 4—figure supplement 1.G-V and SSI relationship data were fitted by a Boltzmann curve. V_1/2_ provides the midpoint voltage of the calculated curve (in mV) and Vc the unit-less slope, with standard error of the mean (SEM). Right column shows peak conductance after toxin treatment as a fraction of untreated peak conductance with the upper and lower bounds of the 95% confidence interval in parentheses, reflecting the data displayed in the dot plots.**DOI:** http://dx.doi.org/10.7554/eLife.10960.012 10.7554/eLife.10960.013Figure 4—source data 2.Table providing values for fits of the data presented in Figure 4—figure supplement 2.G-V and SSI relationship data were fitted by a Boltzmann curve. _1/2_ provides the midpoint voltage of the calculated curve (in mV) and Vc the unit-less slope, with standard error of the mean (SEM). Right column shows peak conductance after toxin treatment as a fraction of untreated peak conductance with the upper and lower bounds of the 95% confidence interval in parentheses, reflecting the data displayed in the dot plots.**DOI:** http://dx.doi.org/10.7554/eLife.10960.013

Although previous work has postulated the involvement of the domain II (DII) S5-S6 pore loop as the region responsible for forming an inter-subunit disulfide bond between particular Na_v_ channel isoforms and β2 or β4 (Chen et al., 2012; Gajewiak et al., 2014), the precise residue has remained elusive. To explore the possibility of an hβ2 anchoring point in this region, we individually replaced each of the three cysteines found here (^910^Cys, ^912^Cys, and ^918^Cys) with Ser. When expressed without or with WT hβ2, the C910S mutant exhibits the same degree of inhibition by 100 nM ProTx-II, indicating that the protective effect of hβ2 is lost and that ^910^Cys is a critical residue for hβ2 binding (Figure 4a–b, Figure 4—source data 1 and Figure 4—figure supplement 1). In contrast, the C918S mutant retains protection from 100 nM ProTx-II by the β-subunit. The C912S mutant displays a split toxin-sensitive population that is consistently observed throughout multiple oocyte batches. One fraction of experiments reveals hNa_v_1.2 current inhibition as though no hβ2 is present whereas another displays protection against 100 nM ProTx-II, similar to the WT channel and C918S mutant (Figure 4a). To verify the presence of hβ2, oocytes were collected after recording and checked for expression by Western blot (see Figure 4b), thereby indicating that the observed loss-of-protection effects indeed relate to the hNa_v_1.2 mutation.

**Figure 4—figure supplement 1.**
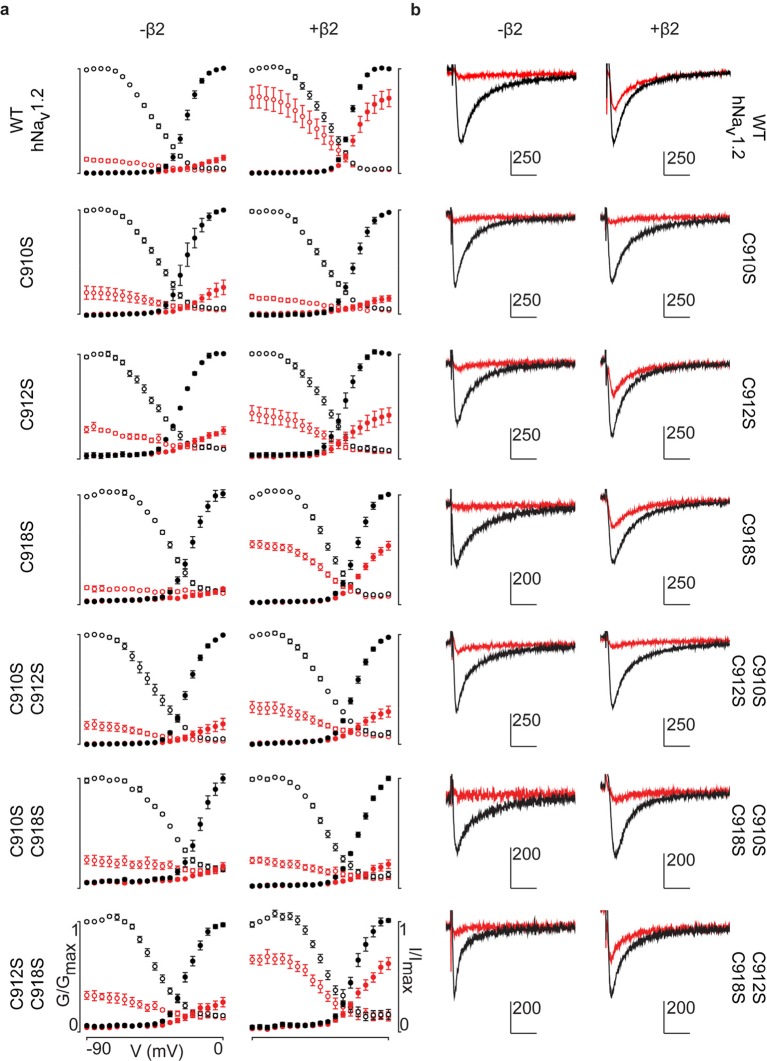
G-V and SSI relationships for hNa_v_1.2 mutants. **a**) G-V (filled circles) and SSI (open circles) curves are plotted for each of the hNa_v_1.2 Cys to Ser mutants with and without hβ2. Black indicates values before the addition of 100 nM ProTx-II,red shows values after toxin treatment. In the presence of hβ2, the WT hNav1.2, the C918S mutant, and the C912S C918S all show a diminished susceptibility to ProTx-II whereas the C912S mutant shows a mixed phenotype. **b**) Sample traces illustrating the effect of 100 nM ProTx-II on hNa_v_1.2 with and without hβ2 after depolarization to -15 mV from -90 mV. Degree of inhibition reflects the G-V and SSI data shown in a). Horizontal arm of scale bar is 10 ms and the vertical arm is in nA. **DOI:** http://dx.doi.org/10.7554/eLife.10960.014

**Figure 4—figure supplement 2.**
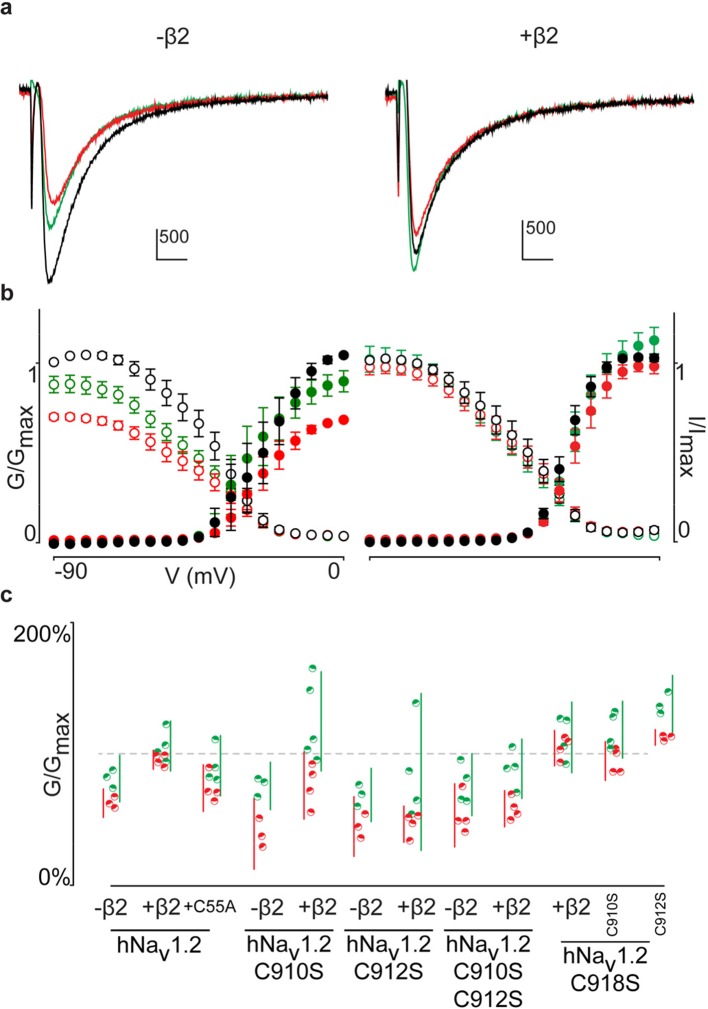
Activity of the μO§-conotoxin GVIIJ is affected by hβ2. (**a**) Representative traces of 5 μM GVIIJ affecting hNa_v_1.2-mediated currents with and without hβ2 in Xenopus oocytes. Control traces are shown in black, red after treatment with 5 μM GVIIJ, and green after a 5-min washout with recording solution. Horizontal bar is 10 ms and vertical bar measures current in nA. The data illustrate that 5 M GVIIJ can partially block hNav1.2 only when hβ2 is not present. (**b**) G-V (filled circles) and SSI (open circles) relationships detailing the effect of 5 M GVIIJ on hNav1.2 with and without hβ2 across a wide voltage range. Color coding is the same as in (**a**). (**c**) Dot plot showing the effect of GVIIJ on hNa_v_1.2 Cys to Ser mutants. Red circles indicate fraction of peak control conductance after addition of 5 μM GVIIJ while green circles represent the same after a 5-min washout. Vertical bars show 95% confidence intervals for color-matched circles. The data clearly illustrate a reduced sensitivity to GVIIJ in the presence of hβ2; however, the results are variable as discussed in the text and led us to pursue ensuing experiments with ProTx-II. **DOI:** http://dx.doi.org/10.7554/eLife.10960.015

Altogether, these results hint towards a possibility of mutation-induced shifts in intra-subunit disulfide bond formations, a plausible scenario given the close vicinity of ^910^Cys, ^912^Cys, and ^918^Cys in hNa_v_1.2. To evaluate this hypothesis, we constructed a set of double Cys to Ser mutations (C910S C912S, C910S C918S, and C912S C918S) and examined the effects on ProTx-II susceptibility without and in the presence of hβ2 (Figure 4a–b, Figure 4—source data 1 and Figure 4—figure supplement 1). According to previous work with rNa_v_1.2a, mutating ^912^Cys or ^918^Cys results in a bridge between ^910^Cys and the remaining intact cysteine (Zhang et al., 2015). These experiments were carried out without a β2 subunit and with GVIIJ (Gajewiak et al., 2014), a unique μO§-conotoxin that directly competes for binding to ^910^Cys; however, the rest of its binding site remains unexplored. In our hands, high concentrations of GVIIJ are needed to achieve an effect, and efficacy towards hNa_v_1.2 is augmented in case of the C910S mutation (Figure 4—source data 2, Figure 4—figure supplement 2), two complicating factors that made us revert to ProTx-II which is more potent and has clearly delineated binding sites within Na_v_1.2. Our experiments on these double mutants show a much clearer picture wherein hβ2 only protects against ProTx-II when ^910^Cys is intact: the C912S C918S mutant still has lowered toxin susceptibility whereas C910S C912S and C910S C918S are completely inhibited by 100 nM ProTx-II, suggesting that these mutants no longer bind hβ2 (Figure 4a–b, Figure 4—figure supplement 1). Altogether, these results indicate that ^910^Cys is the disulfide bond partner of ^55^Cys in hβ2, while ^912^Cys and ^918^Cys could form an intra-subunit bridge. At first sight, the C912S data which show the split population appear in conflict with this interpretation. However, it is conceivable that losing the ^912^Cys- ^918^Cys bond results in the formation of a non-native bond between ^910^Cys and ^918^Cys (Figure 4c). Indeed, when ^918^Cys is mutated in addition to ^912^Cys, ^918^Cys again allows toxin protection by hβ2 similar to WT, suggesting it has become available again. The data also indicate that a non-native disulfide bond between ^910^Cys and ^912^Cys may not occur in the C918S mutant, likely because such a Cys-Val-Cys disulfide bond could be too constrained. Importantly, hβ2 cannot protect the C910S C918S or the C910S C912S mutants from 100 nM ProTx-II, indicating that neither ^912^Cys nor ^918^Cys can compensate for the loss of ^910^Cys (Figure 4c).^918^

**Figure 5.**
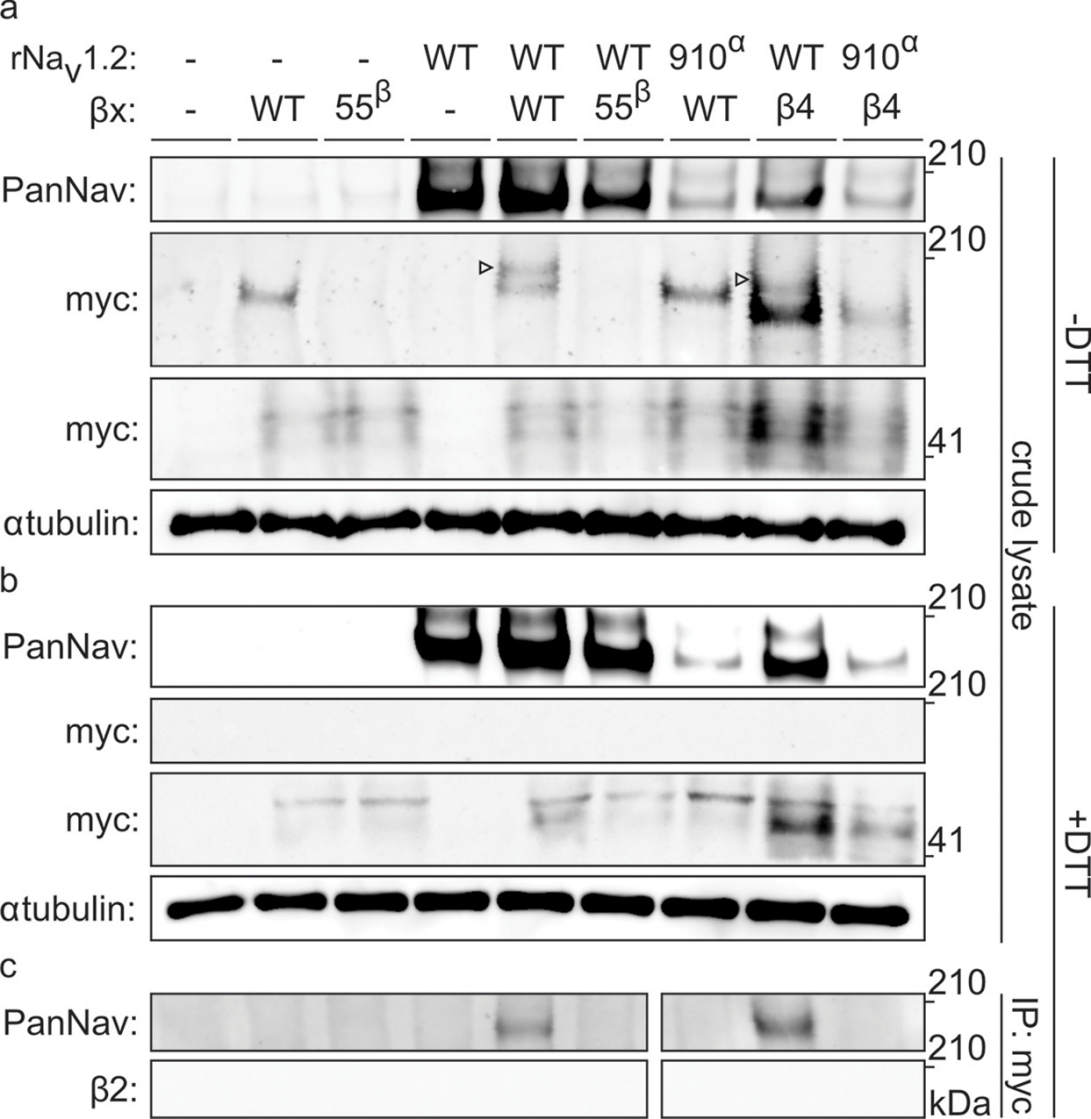
Biochemical verification of the β2 and β4 disulfide bond with ^910^Cys in Na_v_1.2. Top rows indicate particular Na_v_ channel and β-subunit constructs with which oocytes were injected. WT proteins are indicated as such whereas mutants are noted with a residue number; a superscript letter symbolizes in which partner of the complex pair the mutation is found. rβ2 was loaded in each case except in the two rightmost lanes, where rβ4 was used. Labels on left column indicate which antibody was used to immunoblot the associated slice. (**a**) Western blot run under non-reducing conditions reveals a fraction of rβ2 migrating with WT rNa_v_1.2a but not after selective cysteine substitution. Crude lysate from injected oocytes was run on a protein gel and probed for both the channel and the myc-tag of the β-subunit. The top PanNav slice and bottom myc slice show the expression of the respective proteins in lysates from oocytes injected with the indicated constructs. Even though equal quantities of protein was loaded in each lane (10 μg), the C910S channel mutant expresses less bountifully than the WT channel and as a result shows a weaker signal. The WT β-subunit can form redox-sensitive multimers that migrate at an apparent mass similar to that of the Na_v_ channel. Mutation of ^55^Cys prevents multimer formation and disappearance of the high molecular weight band. Open arrows identify bands representing Na_v_ channel-bound β-subunit and aid in distinguishing them from the multimeric β-subunit. Substituting ^910^Cys within rNa_v_1.2a with Ser causes a loss of the channel-bound β-subunit band. rβ4 also binds to WT rNav1.2a, as evidenced by a second band, and no longer interacts with the C910S mutant. (**b**) Addition of DTT prevents both binding of rβ2 to the channel and formation of β-subunit multimers. The absence of myc signal at the same apparent weight as rNa_v_1.2a indicates that binding to the channel is sensitive to reduction. In all cases, α-tubulin was used as a loading control. (**c**) WT rNa_v_1.2a co-immunoprecipitates with rβ2 and rβ4. The β-subunit was pulled down with an antibody directed against a C-terminal myc-tag and treated with DTT before loading onto the gel. The channel is present only when the WT channel is co-expressed with the WT β-subunit. **DOI:** http://dx.doi.org/10.7554/eLife.10960.016

To biochemically verify that Na_v_1.2 and β2 are covalently bound, we expressed the closely related and well-expressing rat variants (∼99% sequence identity) in oocytes and immunoprecipitated the rNa_v_1.2a/rβ2 complex. It is worth noting that WT myc-tagged rβ2 can form higher-order oligomers under non-reducing conditions, which may complicate the interpretation of immunoblots (Figure 5—figure supplement 1). However, these oligomers disappear upon removal of ^55^Cys, further highlighting the reactivity of this residue. Notwithstanding this phenomenon, probing crude cell lysate uncovers a clear co-migration of myc-tagged rβ2 with rNa_v_1.2a under non-reducing conditions, since co-expression yields a distinct myc-stained band above the highest rβ2 oligomer (Figure 5a). Substitution of either ^55^Cys in rβ2 or ^910^Cys in rNa_v_1.2a with Ser results in the loss of this covalent complex. Furthermore, the presence of DTT completely abolishes the rβ2-rNa_v_1.2a complex as well as the rβ2 oligomers (Figure 5b). Thus, these results confirm our toxin experiments and suggest the formation of a disulfide bond between ^910^Cys in the DII S5-S6 linker of Na_v_1.2 and ^55^Cys in β2. In addition to investigating the interaction of rNa_v_1.2a and rβ2 in crude lysate from injected oocytes, we also pulled down rβ2 with an antibody directed against the C-terminal myc-tag and treated with DTT before loading onto a tris-acetate gel (Figure 5c). In this experiment, we observe that WT rNa_v_1.2a is present only when co-expressed with WT rβ2. Moreover, both ^910^Cys within the channel and ^55^Cys in rβ2 are required for co-immunoprecipitation, thereby pointing to their role in forming a disulfide bond between both subunits (Figure 3, Figure 4).

**Figure 5—figure supplement 1.**
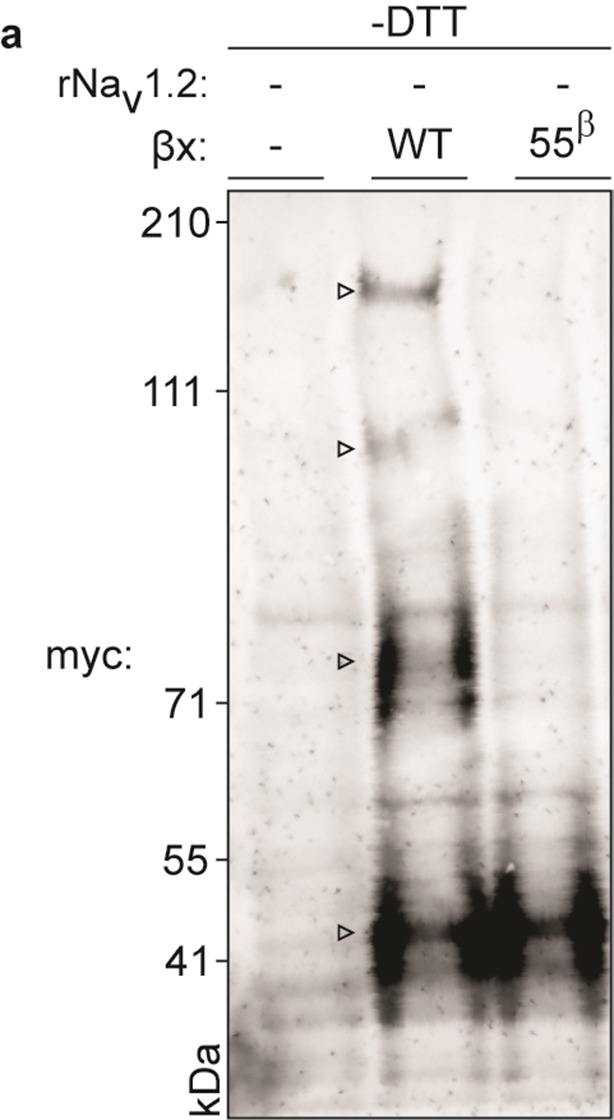
Biochemical assessment of rβ2 oligomer formation. Figure represents extended data found in Figure 5. WT myc-tagged rβ2 can form higher-order oligomers under non-reducing conditions (indicated with an open arrow in the middle lane). These oligomers disappear upon removal of ^55^Cys (right lane), further highlighting the reactivity of this residue. Left lane represents lysate from an uninjected oocyte. **DOI:** http://dx.doi.org/10.7554/eLife.10960.017

**Figure 6.**
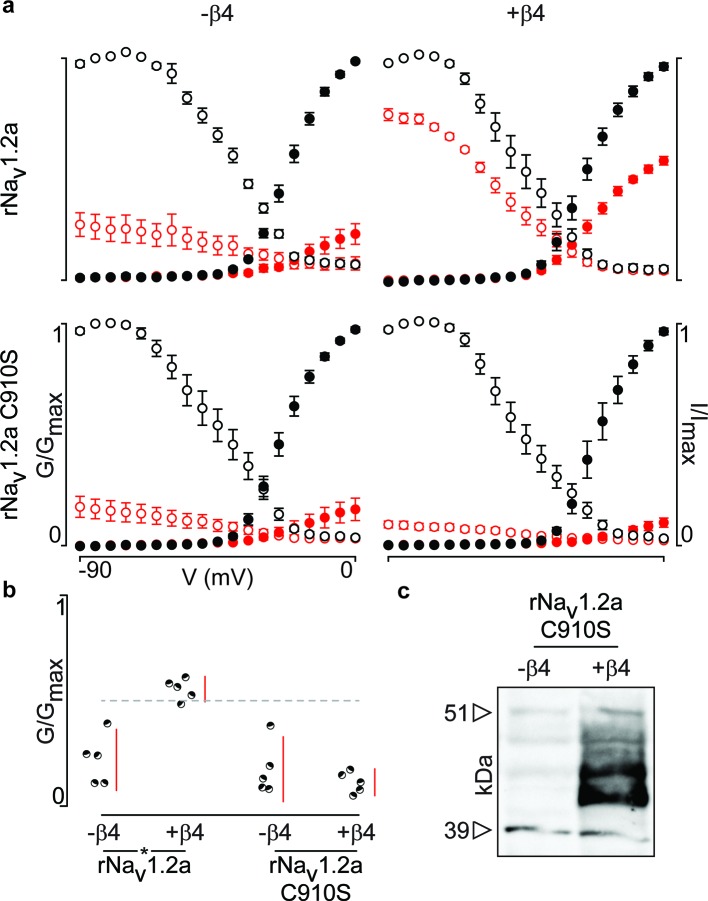
Mutation of ^910^Cys in rNa_v_1.2a disrupts rβ4 influence on ProTx-II effect. (**a**) Replacement of ^910^Cys with Ser in rNa_v_1.2a impairs the ability of rβ4 to protect against 100 nM ProTx-II inhibition in a manner similar to that of hβ2. Co-expression of the rNa_v_1.2a isoform with rβ4 minimizes ProTx-II inhibition compared to the channel expressed alone. Graphs compare G-V and SSI relationships, in filled and open circles, respectively. Black color is used for values before the addition of 100 nM ProTx-II and red after. (**b**) Dot plot comparing the degree of inhibition by 100 nM ProTx-II as a fraction of the pre-toxin peak current. rβ4 confers a high level of a protection against ProTx-II inhibition which is absent in the C910S mutant. (**c**) Western blot directed against the C-terminal myc-tag of rβ4 demonstrating its presence in the oocytes measured. **DOI:** http://dx.doi.org/10.7554/eLife.10960.018 10.7554/eLife.10960.019Figure 6—source data 1.Table providing values for fits of the data presented in Figure 6.G-V and SSI relationship data were fitted by a Boltzmann curve. V_1/2_ provides the midpoint voltage of the calculated curve (in mV) and Vc the unit-less slope, with standard error of the mean (SEM). Right column shows peak conductance after toxin treatment as a fraction of untreated peak conductance with the upper and lower bounds of the 95% confidence interval in parentheses, reflecting the data displayed in the dot plots.**DOI:** http://dx.doi.org/10.7554/eLife.10960.019

Next, we were curious to learn whether ^910^Cys also constitutes an inter-subunit anchoring point for ^58^Cys in the β4-subunit. To examine this notion, we measured ProTx-II susceptibility of rNa_v_1.2a and its C910S mutant expressed in oocytes without or with the rβ4-subunit (Figure 6a,b, Figure 6—source data 1). Analogous to hβ2, we observe rβ4 protein production in oocytes (Figure 6c) where it is able to influence the degree of rNa_v_1.2a current inhibition by ProTx-II. In particular, 100 nM ProTx-II reduces rNa_v_1.2a conductance to ∼22% of peak whereas the current remaining in the presence of rβ4 is ∼55% of peak conductance. Other gating parameters such as G–V and channel availability relationships are unaffected. In case of the C910S mutant, the presence of rβ4 no longer decreases ProTx-II efficacy (from ∼22% current inhibition to ∼17%, respectively, Figure 6—source data 1), thus illustrating the likely role of rNa_v_1.2a ^910^Cys in forming a disulfide bond with rβ4 ^58^Cys. In concert, (co-)immunoprecipitation experiments with rNa_v_1.2a and rβ4 expressed in oocytes indicate that both partners are covalently bound and that mutating ^910^Cys in the channel indeed disrupts the disulfide bond (Figure 5).

**Figure 7.**
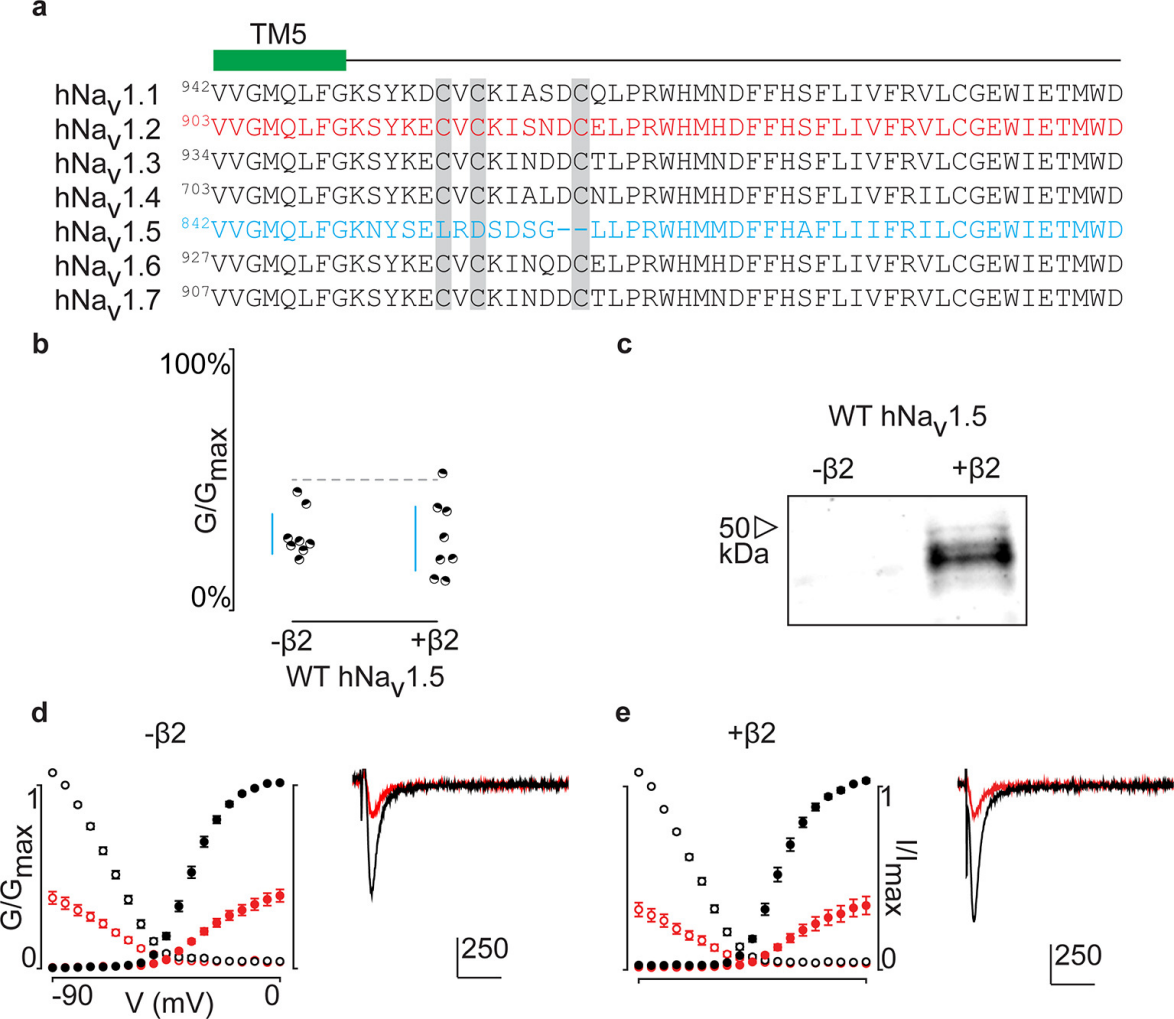
ProTx-II inhibits hNa_v_1.5 in the presence of hβ2. (**a**) Sequence alignment comparing TTX-sensitive hNa_v_ channels in the beginning of the S5-S6 (SS1) loop. Green bar indicates the C-terminal portion of the DII transmembrane segment 5 (TM5), and gray background highlights the conserved cysteine triad. The hNa_v_1.2 amino acid sequence is shown in red and hNa_v_1.5 in blue. Residue number of the N-terminal Val for each channel is superscripted. (**b**) Dot plot showing that hNa_v_1.5 is not protected against inhibition by 100 nM ProTx-II upon co-expressing hβ2. Black circles are individual oocytes of which sodium currents were measured at peak conductance and blue bars show 95% confidence interval. (**c**) Western blot probing for the C-terminal myc-tag of hβ2 reveals its presence in whole cell oocyte fractions. hNa_v_1.5 sees no change in 100 nM ProTx-II effect in the presence of hβ2. (**d, e**) G-V (filled circles) and SSI (open circles) relationships for hNa_v_1.5 with and without hβ2. Black is used for values before ProTx-II addition and red after toxin application. Also shown are representative traces illustrating the lack of effect of hβ2 on hNav1.5 susceptibility to 100 ProTx-II. Horizontal bar indicates 10 ms; vertical bar represents current in nA, with the provided magnitude. **DOI:** http://dx.doi.org/10.7554/eLife.10960.020 10.7554/eLife.10960.021Figure 7—source data 1.Table providing values for fits of the data presented in Figure 7.G-V and SSI relationship data were fitted by a Boltzmann curve. V_1/2_ provides the midpoint voltage of the calculated curve (in mV) and Vc the unit-less slope, with standard error of the mean (SEM). Right column shows peak conductance after toxin treatment as a fraction of untreated peak conductance with the upper and lower bounds of the 95% confidence interval in parentheses, reflecting the data displayed in the dot plots.**DOI:** http://dx.doi.org/10.7554/eLife.10960.021

Aligning the primary sequences of all tetrodotoxin (TTX)-sensitive hNa_v_channel isoforms (Na_v_1.1–1.7) reveals that the cardiac hNa_v_1.5 channel lacks the cysteine triad while the flanking sequences are highly conserved (Figure 7a). Subsequently, it seems unlikely that hNa_v_1.5 forms a covalent bond with hβ2, unless it occurs via a distinct site. However, when applying 100 nM ProTx-II to hNa_v_1.5 without and with the β-subunit, we observe no effects on gating or protection against the toxin, suggesting that hβ2 may not modulate this channel isoform (Figure 7b–e, Figure 7—source data 1). It is worth mentioning that hNa_v_1.8 and hNa_v_1.9, two TTX-resistant isoforms that are evolutionary related to hNa_v_1.5, also lack the cysteine triad and may therefore not interact with hβ2 via a disulfide bond.

### Positioning β2 in relation to Na_v_1.2

**Figure 8.**
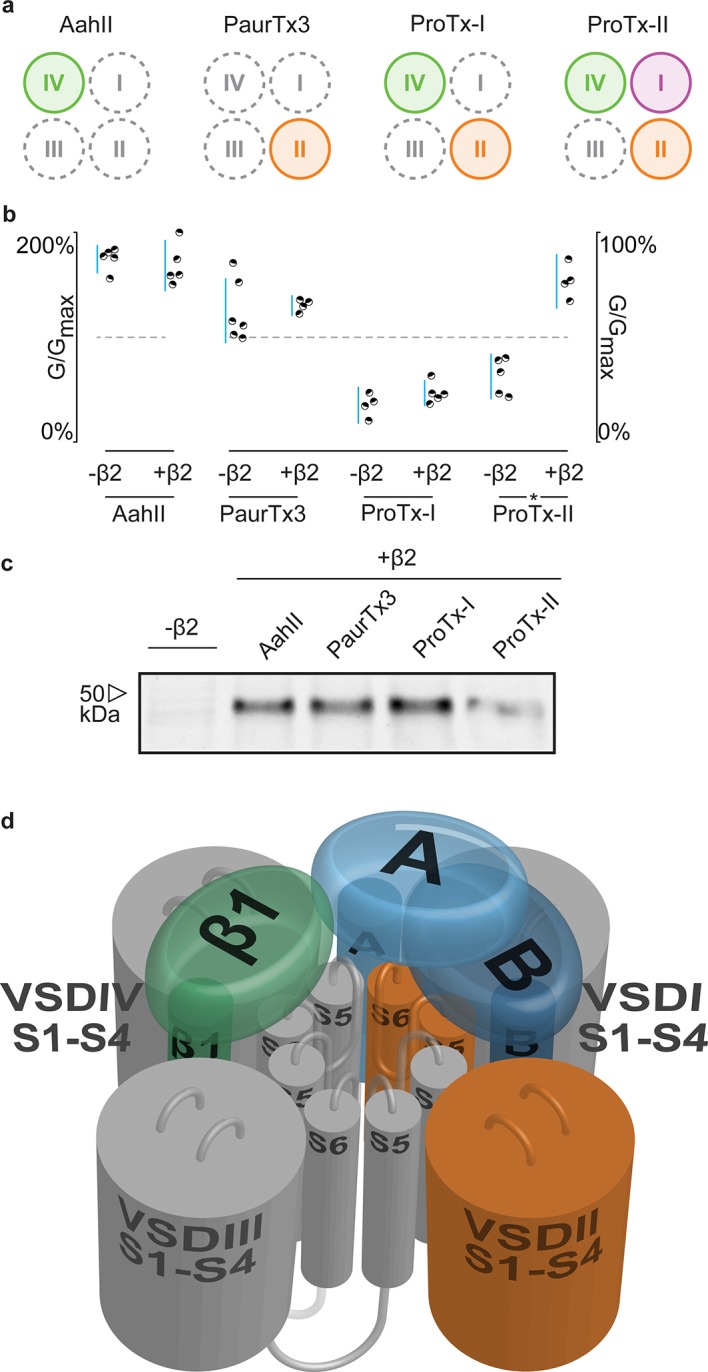
hβ2 influences hNa_v_1.2 VSDI toxin pharmacology. (**a**) Cartoon illustrating the binding site of AaHII, PaurTx3, ProTx-I, and ProTx-II within the VSDs of Na_v_1.2. Binding to a particular VSD is indicated by coloration. (**b**) Dot plot showing percent of change in peak conductance for hNa_v_1.2 after treatment by each toxin without or in the presence of hβ2. Blue bars represent 95% confidence interval and statistical significance (p<0.01) is noted by an asterisk. **c**) Western blot probing for the C-terminal myc-tag of hβ2, showing its presence in each experimental condition except the negative control. (**d**) Shown is a Na_v_ channel illustration consisting of the four VSDs I-IV (S1-S4) and the corresponding pore-forming regions (S5-S6) in grey (DI, III, and IV) or orange (DII) in a clockwise orientation which fits with the data reported here. Such an orientation places a S1-S4 voltage sensor of one domain adjacent to the S5-S6 region of the next. A/B (shades of blue) depicts a putative location of hβ2 in the complex where the subunit can interact with the S5-S6 pore region of DII as well as occlude the ProTx-II binding site on VSDI. In contrast, hβ1 (green) may position itself between VSDIV and VSDIII where it can interact with the S5-S6 region of DI and influence VSDIV movement to alter channel fast inactivation. **DOI:** http://dx.doi.org/10.7554/eLife.10960.022 10.7554/eLife.10960.023Figure 8—source data 1.Table providing values for fits of the data presented in Figure 8 and Figure 8—figure supplement 1.G-V and SSI relationship data were fitted by a Boltzmann curve. V_1/2_ provides the midpoint voltage of the calculated curve (in mV) and Vc the unit-less slope, with standard error of the mean (SEM). Right column shows peak conductance after toxin treatment as a fraction of untreated peak conductance with the upper and lower bounds of the 95% confidence interval in parentheses, reflecting the data displayed in the dot plots.**DOI:** http://dx.doi.org/10.7554/eLife.10960.023

Having identified ^910^Cys within the DII S5-S6 loop of hNa_v_1.2 and ^55^Cys within hβ2 as inter-subunit disulfide bond partners, we sought to find evidence for a potential locus of the β-subunit in relation to the channel. Although hβ2 seems to be anchored to the DII pore forming region, ProTx-II was previously shown to target VSDs I, II, and IV (Bosmans et al., 2008), suggesting that the protective effect of hβ2 is through occlusion of the ProTx-II binding site in one or more of these regions. In order to determine which one, we assembled a set of animal toxins that, together, interact with VSDI, II, and IV in Na_v_1.2 (Bosmans et al., 2008), and tested whether or not their function is influenced by hβ2 (Figure 8a). A previous study in which rNa_v_1.2a S3b-S4 paddle loops from each domain (I-IV) were transplanted into a homotetrameric K_v_ channel to identify the VSDs with which toxins interact (Bosmans et al., 2008), found that the tarantula toxin PaurTx3 and scorpion toxin AahII (Martin et al., 1987) exclusively target VSDII and VSDIV, respectively. In addition, the tarantula toxins ProTx-I and ProTx-II both interact with the voltage sensor in DII and DIV, whereas ProTx-II also binds to DI with high affinity. Here, we tested these four toxins on hNa_v_1.2 without and with hβ2 to determine if the presence of the subunit impacts toxin function. Aside from ProTx-II, we observe no significant difference in PaurTx3, AahII, or ProTx-I effect which suggests that hβ2 does not impede binding of these toxins to VSDII and VSDIV (Figure 8b–c, Figure 8—source data 1 and Figure 8—figure supplement 1). Therefore, we speculate that hβ2 primarily influences VSDI and as such, is located near this region (Figure 8d). Since μO§-conotoxin GVIIJ function is also influenced by hβ2 (Gajewiak et al., 2014), it will be interesting to investigate a possible VSDI binding site for this toxin. While our data are limited by the absence of VSDI- and VSDIII-specific toxins and do not exclude the possibility of non-overlapping binding sites for a particular toxin and hβ2 on the same VSD, or of direct competition for binding to the DII S5-S6 loop, they may yet prove valuable as insights into hβ2 function accrue.

**Figure 8—figure supplement 1.**
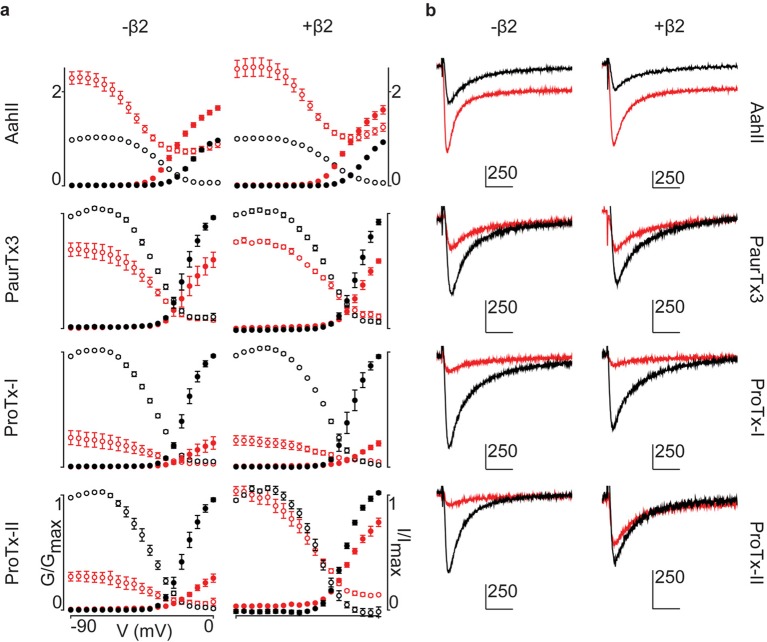
Activity of spider and scorpion toxins on hNa_v_1.2. G-V and SSI relationships for hNav1.2, with or without hβ2, treated with toxins (100 nM) with known binding sites. (**a**) G-V and SSI curves are provided for each of the four toxins tested. Black indicates control values and red illustrates toxin effect. Filled circles are used for G-V curves and open circles for SSI. (**b**) Representative traces after depolarization to -15 mV from -90 mV for each toxin tested on hNa_v_1.2 with black indicating pre-toxin currents and red showing currents after toxin application. Horizontal scale bar indicates 10 ms whereas vertical bar shows current magnitude in nA, with scale given by the attached number. **DOI:** http://dx.doi.org/10.7554/eLife.10960.024

## Discussion

The goal of this study was to explore the interaction of β2 with Na_v_1.2 and identify anchoring residues in both partners which will help orient functional motifs within their extracellular domains. Although we obtained the first crystal structure of the hβ4 extracellular region (Gilchrist et al., 2013), the second reported structure of the corresponding hβ3 domain (Namadurai et al., 2014) highlights the unique character of each β-subunit isoform. Since the ability to compare the distinct organizational features of β-subunits will provide valuable information as to their difference in action, we now obtained detailed structural information for the extracellular domain of hβ2 at a resolution of 1.35Å (Figure 1a–b, Figure 1—source data 1). We identified a flexible loop formed by ^72^Cys and ^75^Cys that is a unique feature among β-subunits but with a function that has yet to be elucidated (Figure 1c, Figure 2, and Figure 3). Moreover, hβ2 contains a Cys at position 55 that, when mutated, disrupts the influence of hβ2 on hNa_v_1.2 toxin pharmacology (Figure 3b–d). Next, we combined mutagenesis and biochemical studies with spider and scorpion toxins that target specific VSDs within Na_v_1.2 (Gilchrist et al., 2014; Bosmans et al., 2008) to probe the interaction with hβ2. As a result, we found that ^55^Cys forms a distinct disulfide bond with ^910^Cys located in the domain II S5-S6 loop of hNa_v_1.2, thereby revealing a 1:1 stoichiometry (Figure 4a–b, Figure 4—source data 1 and Figure 4—figure supplement 1). We also exploited this toxin-reporter approach to investigate the possibility of intra-subunit disulfide bond formations between ^910^Cys, ^912^Cys, and ^918^Cys in hNa_v_1.2, three reactive residues that are in close vicinity to each other. The outcome of these experiments indicates that ^912^Cys and ^918^Cys form an intra-subunit bridge in WT hNa_v_1.2 (Figure 4). When mutating ^918^Cys, hβ2 still interacts with ^910^Cys whereas substituting ^912^Cys suggests the possibility for bond formation between ^910^Cys and ^918^Cys (Figure 4c).

Interestingly, hNa_v_1.5 lacks these three cysteines (Figure 7a) and as a result, ProTx-II is equipotent without or in the presence of hβ2 (Figure 7b–e, and Figure 7—source data 1) This outcome provides evidence that this particular β-subunit may not modulate hNa_v_1.5 in heterologous systems or that binding occurs through a different mechanism in native tissues. In concert, immunocytochemical studies in the heart and electrophysiological measurements in mammalian cell lines or oocytes disagree on whether hβ2 can modulate hNa_v_1.5 function (Johnson and Bennett, 2006; Zimmer and Benndorf, 2002, 2007; Dhar Malhotra et al., 2001; Maier et al., 2004). As opposed to native cardiomyocytes, hNa_v_1.5 may not associate with hβ2 upon heterologous expression, a hypothesis that is supported by co-localization experiments in HEK293 cells where hNa_v_1.5 and hβ2 were mainly found in the endoplasmatic reticulum or plasma membrane, respectively (Zimmer et al., 2002).

So far, none of the hβ2 mutations implicated in disorders have been found close to ^55^Cys, as they are located either in the signal peptide or near the transmembrane region (Li et al., 2013; Medeiros-Domingo et al., 2007; Tan et al., 2010; Baum et al., 2014; Watanabe et al., 2009). In contrast, amino acid substitutions in the DII S5-S6 pore loop of particular Na_v_ channel variants are linked to diseases. For example, more than 30 mutations have been identified within this region in hNa_v_1.1, several of which relate to Dravet syndrome (Claes et al., 2009). This includes C927F (corresponding to ^918^Cys in hNa_v_1.2), and other variants which introduce an additional Cys that may interfere with local disulfide bond pattern formation. Therefore, it is conceivable that DII S5-S6 linker mutations may affect channel interactions with hβ2 or hβ4.

Collectively, our results uncover the disulfide link between hβ2 and hNa_v_1.2 which opens up the possibility to assign a potential orientation of this subunit in relation to the channel and provide an experimental basis for future docking efforts (Figure 8d). hβ2 may position itself in the gaps between VSDs where it can interact with a voltage sensor as well as anchor to a pore-forming region. One important observation from bacterial Na_v_ channel crystal structures (Payandeh et al., 2012; Payandeh et al., 2011; Shaya et al., 2014; Zhang et al., 2012) is the domain-swapped architecture of the channel in which the S1-S4 VSD within one subunit is located adjacent to the S5-S6 segments of the next subunit. Such a structural clockwise arrangement has also been consistently observed in prokaryotic and mammalian K_v_ channels (Long et al., 2007; Jiang et al., 2003). Since hNa_v_ channels may be organized in a similar fashion, the determinants of hβ2-subunit sensitivity can be located in multiple Na_v_ channel domains. For example, we found that hβ2 binds to ^910^Cys in the S5-S6 loop of DII and influences ProTx-II interaction with VSDI. Although it is challenging to pinpoint its precise location, our data suggest that hβ2 is positioned in the cleft between VSDIV and VSDI or VSDI and VSDII where it is presented with an opportunity to interact with both determinants to influence ProTx-II action (Figure 8d). In contrast, the consistent picture emerging from the literature is that Na_v_ channel fast inactivation and voltage-dependence of activation changes substantially when co-expressed with the β1-subunit in heterologous systems (Chen and Cannon, 1995; McCormick et al., 1998; Qu et al., 1999; Isom et al., 1995b). Given the pivotal role of VSDIV in channel fast inactivation (Bosmans et al., 2008; Capes et al., 2013; Chanda and Bezanilla, 2002; Horn et al., 2000; Sheets et al., 1999), β1 may be positioned close to VSDIV where it can also interact with the extracellular S5-S6 pore-loop within DI (Figure 8d), a presumed interacting region (Qu et al., 1999; Makita et al., 1996). Altogether, this model suggests that particular Na_v_ channel isoforms can interact simultaneously with a β1- and β2-subunit (Calhoun and Isom, 2014). Future experiments will further investigate the possible locations of β3 and β4 in this model as well as allow the incorporation of mutational effects which is essential to uncover the contribution of β-subunit mutations to human disorders or contribute to small molecule screening efforts geared towards disrupting or facilitating subunit interactions.

## Materials and methods

### Production of the hβ2 extracellular domain

Human β2 (residues 30–153) was cloned into a modified pET28 vector (pET28HMT) (Van Petegem et al., 2004). Mutations (C55A and C55/72/75A) were introduced using the Quikchange kit from Agilent Technologies (USA) according to the manufacturer’s instructions. Proteins were expressed at 18°C in *E. coli* Rosetta (DE3) pLacI strains (Novagen, USA), induced at an OD600 of ∼0.6 with 0.4 mM IPTG, and grown overnight prior to harvesting. Cells were lysed via sonication in buffer A (250 mM KCl and 10 mM HEPES at pH 7.4), supplemented with 25 µg/ml DNaseI and 25 µg/ml lysozyme. After centrifugation, the supernatant was applied to a PorosMC column (Tosoh Biosep, USA), washed with buffer A plus 10 mM imidazole, and eluted with buffer B (250 mM KCl plus 500 mM imidazole pH 7.4). The protein was dialyzed overnight against buffer A and cleaved simultaneously with recombinant TEV protease. Next, the samples were run on another PorosMC column in buffer A, and the flowthrough was collected and dialyzed against buffer C (10 mM KCl plus 10 HEPES at pH 7.4), applied to a HiloadQ column (GE Healthcare, USA), and eluted with a gradient from 0% to 30% buffer D (1 M KCl plus 10 mM HEPES at pH 7.4). Finally, the samples were run on a Superdex200 (GE Healthcare, USA) gel filtration column in buffer A. The protein samples were exchanged to 50 mM KCl plus 10 mM HEPES (pH 7.4), concentrated to ∼5 mg/ml using Amicon concentrators (3K MWCO; Millipore USA), and stored at −80°C.

### Crystallization, data collection, and structure solution

Crystals were grown using the hanging-drop method at 4°C. Both C55A and C55/72/75A were crystallized in 0.1 M Tris (pH 8), 15–20% (w/v) PEG 6000. Crystals were ﬂash-frozen after transfer to the same solution supplemented with 30% glycerol. The data used to solve the final structures were collected at the Canadian Light Source (Saskatoon) beamline 08ID-1 and datasets were processed using XDS (Kabsch, 2010) and HKL3000 (Minor et al., 2006). A search model was created by using only β-strands from PDB 4 MZ2, and with all side chains truncated to alanine. Molecular replacement was performed using Phaser (McCoy et al., 2007), yielding poor initial phases which were improved via autobuilding in ARP/wARP (Langer et al., 2008). The model was completed by successive rounds of manual model building in COOT (Emsley et al., 2010) and refinement using Phenix (Adams et al., 2010). A simulated annealing composite omit map was calculated with CNS (Brünger et al., 1998) to verify the absence of residual model bias. ^85^Met was found to be in a disallowed region of the Ramachandran plot in both structures. All structure ﬁgures were prepared using PYMOL (DeLano Scientiﬁc, San Carlos, USA).

### Accession codes

Coordinates and structure factors have been deposited in the Protein Data Bank under accession codes 5FEB (C55A) and 5FDY (C55/72/75A).

### Toxin acquisition and purification

ProTx-I and ProTx-II were acquired from Peptides International (USA), PaurTx3 from Alomone Labs (Israel). AaHII from *Androctonus australis* hector venom was purified as described (Martin et al., 1987). Toxins were kept at -20°C and aliquots were dissolved in appropriate solutions containing 0.1% BSA.

### Two-electrode voltage-clamp recording from *Xenopus* oocytes

The DNA sequence of hNa_v_1.2 (NM_021007.2), rNa_v_1.2a (NM_012647.1), rβ4 (NM_001008880) and hβ2 (NM_004588.4) (acquired from Origene, USA), as well as their mutants was confirmed by automated DNA sequencing and cRNA was synthesized using T7 polymerase (mMessage mMachine kit, Ambion) after linearizing the DNA with appropriate restriction enzymes. Channels were expressed in *Xenopus* oocytes together with a β-subunit (1:5 molar ratio) and studied following 1–2 days incubation after cRNA injection (incubated at 17°C in 96 mM NaCl, 2 mM KCl, 5 mM HEPES, 1 mM MgCl_2_ and 1.8 mM CaCl_2_, 50 μg/ml gentamycin, pH 7.6 with NaOH) using two-electrode voltage-clamp recording techniques (OC-725C, Warner Instruments) with a 150 μl recording chamber. The data were filtered at 4 kHz and digitized at 20 kHz using pClamp 10 software (Molecular Devices, USA). Microelectrode resistances were 0.5–1 MΩ when filled with 3 M KCl. The external recording solution contained 100 mM NaCl, 5 mM HEPES, 1 mM MgCl_2_ and 1.8 mM CaCl_2_, pH 7.6 with NaOH. The experiments were performed at room temperature (∼22°C) and leak and background conductances, identified by blocking the channel with tetrodotoxin (Alomone Labs, Israel), have been subtracted for all Na_v_ channel currents. All chemicals used were obtained from Sigma-Aldrich (USA) unless indicated otherwise.

### Analysis of channel activity and toxin–channel interactions

Voltage–activation relationships were obtained by measuring steady-state currents and calculating conductance (G), anitted to the data according to: $G/G_{\max}\;=\;{\left(1\;+\;e^{-\mathrm{zF}(V\;-\;V_{1/2})/\mathrm{RT}}\right)}^{-1}\;$ where G/G_max_ is the normalized conductance, *z* is the equivalent charge, V_1/2_ is the half-activation voltage, *F* is Faraday's constant, R is the gas constant and T is temperature in Kelvin. Occupancy of closed or resting channels by ProTx-II and other toxins was examined using negative holding voltages where open probability was very low, and the fraction of uninhibited channels (Fu) was estimated using depolarizations that are too weak to open toxin-bound channels, as described previously. After addition of the toxin to the recording chamber, the equilibration between the toxin and the channel was monitored using weak depolarizations typically elicited at 5 s intervals. Off-line data analysis was performed using Clampfit 10 (Molecular Devices, USA), and Origin 8 (Originlab, USA).

### Qualitative biochemical assessment of hβ2 production in *Xenopus* oocytes

After each electrophysiological experiment, oocytes expressing hNa_v_1.2, hβ2, and the described mutants were washed with ND100 and incubated with 0.5 mg/ml Sulfo-NHS-LC-biotin (Pierce, USA) for 30 min. Oocytes were thoroughly washed again in ND100 before lysis (by pipetting up and down) in 20 μl/oocyte buffer H (1% Triton X-100, 100 mM NaCl, 20 mM Tris-HCl, pH 7.4) plus protease inhibitors (Clontech, USA). All subsequent steps were performed at 4°C. Lysates were gently shaken for 15 min after which they were centrifuged at 16,200x*g* for 3 min. The pellet was discarded and the supernatant (SN) transferred to a fresh 1.l Eppendorf tube. 40 μl of SN was stored at -80°C for later use as the whole cell protein aliquot. 200 μl of hydrophilic streptavidin magnetic beads (New England Biolabs, USA) were then added and the sample shaken gently at 4°C overnight. Beads were washed 6 times with buffer H and resuspended in 40 μl buffer H, after which the biotinylated protein was dissociated from the beads through the addition of 1X LDS loading buffer plus reducing agent (10% 2-ME, 50 mM DTT final conc.) and boiling at 95°C for 5 min to generate the surface protein fraction. All samples were appropriately diluted in buffer H to give equal protein concentrations, as measured by a BCA assay (Pierce, USA). 10 μg of the SN was run on a 10% Tris-Glycine Novex Mini-Gel (Thermo Fisher Scientific, USA) with Tris-Glycine running buffer and analyzed by Western analysis. Nitrocellulose membranes were probed overnight at 4°C with 1:1000 mouse anti-myc antibody as primary (Cell Signaling Technologies, USA) and for 45 min at room temperature with 1:10000 goat anti-mouse HRP-conjugated antibody as secondary (Thermo-Fisher Scientific, USA). Membranes were incubated for 5 min with an enhanced chemiluminescent substrate before imaging.

### Immunoprecipitation of the Na_v_1.2/β2 complex

*Xenopus* oocytes were injected with 5 ng RNA of rβ2, rNa_v_1.2, or both (∼1:5 molar ratio) and incubated for 48 hr at 17°C. 30 oocytes were lysed for each condition, using 20 μl lysis buffer (1x PBS, 1% DDM, 10% glycerol, 1 mM EDTA, 1X protease inhibitor cocktail [Clontech, USA]) per oocyte, and homogenized by passing through a 25-gauge syringe (adapted from [Yu, 2012]). The lysate was rotated for 1 hr at 4°C in a 1.5 ml microfuge tube, after which it was spun for 30 min at 20,000x*g* at 4°C. Subsequent steps were performed at 4°C or on ice and during all rotations the tube was sealed with parafilm to prevent leakage. A fresh pipette tip was gently swirled in the supernatant to remove the bulk of the white goop by adhering to the tip and supernatant was transferred to a new tube, taking care not to disturb the pellet. The new tube was spun 3 min at 20,000x*g* and again was swirled with a fresh pipette tip to remove the white goop, after which the clear supernatant was transferred to a new tube. Protein concentration was assayed using a BCA protein concentration kit (Pierce, USA). 150 μg protein was brought to 150 μl in lysis buffer and 1.5 μg (1.5 μl of 1 mg/ml) anti-myc mouse antibody (Thermo-Fisher Scientific, USA) was added. The tube was rotated overnight at 4°C after which 30 μl protein G-coated magnetic Dynabeads (Thermo-Fisher Scientific, USA) were added and rotated for 4 hr at 4°C. The tube was spun for 1 min at 20,000x*g* and placed on a magnetic rack for 1 min to collect the beads. Supernatant was removed and stored for optimization and troubleshooting purposes, as were subsequent washes. The beads were washed with 200 μl wash buffer (1x PBS, 0.4% DDM, 10% glycerol, 1 mM EDTA) by pipette mixing and then magnetized to collect beads. The wash process was repeated three times. After the third wash, beads were suspended in 100 μl wash buffer, transferred to another tube, spun for 1 min at 20,000x*g*, and magnetized to collect beads. The tube was then spun again for 1 min at 20,000x*g* and magnetized, followed by removal of the last residual fluid. Protein was eluted in 30 μl elution buffer (50 mM glycine, pH 2.8) by incubating for 3 min at room temperature, followed by magnetization and transfer to the final tube. For Western blotting of the immunoprecipitate, 17 μl of eluate was combined with 2.5 μl 10X reducing agent (Thermo-Fisher Scientific, USA) and 6.5 μl 4X LDS sample loading buffer (Thermo-Fisher Scientific, USA), heated for 10 min at 37°C, then loaded onto a 1.0 mm 12-well 3–8% Novex Tris-Acetate pre-cast gel (Thermo-Fisher Scientific, USA). A rabbit anti-PanNav antibody (Alomone labs, Israel) and rabbit anti-hβ2 antibody (Cell Signaling Technologies, USA) were chosen to avoid cross-reaction of the Western blot secondary antibody against the mouse antibody used for immunoprecipitation. For Western blotting, the primary antibodies were used at 1:200 and 1:1000 dilutions, respectively, applied for 1 hr at room temperature and incubated for 45 min at room temperature with a 1:10,000 goat anti-rabbit HRP-conjugated secondary antibody (Thermo-Fisher Scientific, USA).
