# Supplementary material for: Binary architecture of the Nav1.2-β2 signaling complex
Source: eLife. 2016 Feb 19;5:e10960. doi: 10.7554/eLife.10960 (PMC4769172; doi:10.7554/eLife.10960)
Supplement: Figure 1—source data 1. — DOI: http://dx.doi.org/10.7554/eLife.10960.004 [file elife-10960-fig1-data1.doc]

**Table 1** **Data collection and refinement statistics**

|  | | **C55A** | **C55/72/75A** |
| --- | --- | --- | --- |
| **Data collection** |  | |  |
| Space group | *P*2_1_2_1_2_1_ | | *P*1 |
| Cell dimensions   |  | |  |
| *a*, *b*, *c* (Å) | 28.7, 59.4, 71.3 | | 28.4, 36.2, 59.2 |
|     α, β, γ  (°) | 90.0, 90.0, 90.0 | | 89.9, 89.9, 76.4 |
| Resolution (Å) | 45.67 –1.35 (1.43-1.35) ^a, b^ | | 30.0 -1.85 (1.88-1.85) ^a, b^ |
| *R*_sym_ or *R*_merge_  *CC*_1/2_^c^ | 7.1 (193.3)  0.725 | | 10.0 (32.7)  0.845 |
| *I* / σ*I* | 20.36 (1.81) | | 14.94 (2.56) |
| Completeness (%) | 99.4 (96.2) | | 99.0 (90.1) |
| Redundancy | 14.08 (13.8) | | 3.0 (1.6) |
| **Refinement** |  | |  |
| Resolution (Å) | 45.67 –1.35 | | 30-1.85 |
| No. reflections | 27,539 | | 19,296 |
| *R*_work_ / *R*_free_ | 17.22/20.61 | | 15.62/18.53 |
| No. atoms |  | |  |
| Protein  Ligand | 2392  14 | | A: 1066  B: 1065  6 |
| Water | 119 | | 364 |
| *B*-factors |  | |  |
| Protein  Ligand | 29.45  71.39 | | A: 16.5  B: 16.5  37.5 |
| Water | 37.62 | | 28.8 |
| R.m.s. deviations |  | |  |
| Bond lengths (Å) | 0.009 | | 0.004 |
| Bond angles (°) | 0.995 | | 0.648 |

^a^ One crystal for each structure was used for data collection and

structure determination.

^b^ Values in parentheses are for highest-resolution shell.

c Value is for highest-resolution shell.
