# Supplementary material for: Binary architecture of the Nav1.2-β2 signaling complex
Source: eLife. 2016 Feb 19;5:e10960. doi: 10.7554/eLife.10960 (PMC4769172; doi:10.7554/eLife.10960)
Supplement: Figure 3—source data 1. — G-V and SSI relationship data were fitted by a Boltzmann curve. V1/2 provides the midpoint voltage of the calculated curve (in mV) and Vc the unit-less slope, with standard error of the mean (SEM). Right column shows peak conductance after toxin treatment as a fraction of untreated peak conductance with the upper and lower bounds of the 95% confidence interval in parentheses, reflecting the data displayed in the dot plots. DOI: http://dx.doi.org/10.7554/eLife.10960.008 [file elife-10960-fig3-data1.docx]

|  | | | activation | | inactivation | | peak Gafter/peak Gbefore |
| --- | --- | --- | --- | --- | --- | --- | --- |
|  |  |  | V1/2 | Vc | V1/2 | Vc |  |
| hNav1.2 WT | -β2 | before | -20.2 ± 0.2 | 5.5 ± 0.1 | -37.3 ± 0.8 | 7.3 ± 0.6 | 0.17 (0.13, 0.20) |
|  |  | after | -2.3 ± 3.5 | 12.2 ± 0.8 | -44.1 ± 2.2 | 13.3 ± 1.3 |  |
|  | +β2 WT | before | -20.7 ± 0.1 | 5.5 ± 0.1 | -38.0 ± 0.6 | 8.9 ± 0.6 | 0.64 (0.50, 0.79) |
|  |  | after | -17.9 ± 0.6 | 5.9 ± 0.2 | -39.8 ± 1.0 | 9.3 ± 0.6 |  |
|  | +β2  C55A | before | -24.0 ± 0.3 | 5.0 ± 0.1 | -36.8 ± 0.8 | 7.6 ± 0.6 | 0.19 (0.12, 0.26) |
|  |  | after | -12.8 ± 2.7 | 9.1 ± 0.8 | -39.9 ± 1.4 | 9.4 ± 1.0 |  |
|  | +β2  C55S | before | -24.0 ± 0.5 | 5.2 ± 0.1 | -39.7 ± 0.5 | 8.4 ± 0.4 | 0.18 (0.13, 0.22) |
|  |  | after | -12.6 ± 2.1 | 9.0 ± 0.7 | -43.2 ± 1.3 | 11.1 ± 1.0 |  |
|  | +β2  C72A C75A | before | -20.9 ± 0.2 | 5.3 ± 0.1 | -37.7 ± 0.6 | 8.8 ± 0.5 | 0.59 (0.47, 0.70) |
|  |  | after | -18.2 ± 0.6 | 5.9 ± 0.2 | -38.7 ± 0.9 | 8.9 ± 0.6 |  |

**Table 2. Table providing values for fits of the data presented in Fig. 3 and Supplementary File 2.** G-V and SSI relationship data were fitted by a Boltzmann curve. V_1/2_ provides the midpoint voltage of the calculated curve (in mV) and Vc the unit-less slope, with standard error of the mean (SEM). Right column shows peak conductance after toxin treatment as a fraction of untreated peak conductance with the upper and lower bounds of the 95% confidence interval in parentheses, reflecting the data displayed in the dot plots.
