# Supplementary material for: Binary architecture of the Nav1.2-β2 signaling complex
Source: eLife. 2016 Feb 19;5:e10960. doi: 10.7554/eLife.10960 (PMC4769172; doi:10.7554/eLife.10960)
Supplement: Figure 4—source data 1. — G-V and SSI relationship data were fitted by a Boltzmann curve. V1/2 provides the midpoint voltage of the calculated curve (in mV) and Vc the unit-less slope, with standard error of the mean (SEM). Right column shows peak conductance after toxin treatment as a fraction of untreated peak conductance with the upper and lower bounds of the 95% confidence interval in parentheses, reflecting the data displayed in the dot plots. DOI: http://dx.doi.org/10.7554/eLife.10960.012 [file elife-10960-fig4-data1.docx]

|  | | | activation | | inactivation | | peak Gafter/peak Gbefore |
| --- | --- | --- | --- | --- | --- | --- | --- |
|  |  |  | V1/2 | Vc | V1/2 | Vc |  |
| hNav1.2 WT | -β2 | before | -20.7 ± 0.1 | 5.5 ± 0.1 | -43.9 ± 0.8 | 9.3 ± 0.6 | 0.14 (0.05, 0.23) |
|  |  | after | -6.2 ± 4.9 | 11.4 ± 1.4 | -45.8 ± 1.7 | 12.7 ± 1.5 |  |
|  | +β2 | before | -21.7 ± 0.2 | 4.4 ± 0.1 | -39.7 ± 1.0 | 7.8 ± 0.6 | 0.71 (0.47, 0.94) |
|  |  | after | -17.8 ± 0.8 | 5.3 ± 0.3 | -39.7 ± 1.3 | 7.9 ± 0.7 |  |
| C910S | -β2 | before | -20.7 ± 0.4 | 6.0 ± 0.2 | -41.7 ± 0.5 | 9.6 ± 0.5 | 0.16 (0.14, 0.19) |
|  |  | after | 1.3 ± 8.0 | 12.1 ± 1.4 | -46.3 ± 1.2 | 14.9 ± 0.9 |  |
|  | +β2 | before | -18.3 ± 0.2 | 6.0 ± 0.1 | -41.4 ± 0.7 | 9.2 ± 0.5 | 0.17 (0.09, 0.24) |
|  |  | after | 8.9 ± 18.2 | 19.0 ± 3.3 | -44.5 ± 3.2 | 13.2 ± 2.9 |  |
| C912S | -β2 | before | -23.8 ± 0.1 | 6.5 ± 0.1 | -43.5 ± 0.7 | 10.0 ± 0.6 | 0.26 (0.15, 0.37) |
|  |  | after | -4.7 ± 10.3 | 14.3 ± 2.7 | -57.2 ± 5.1 | 16.6 ± 340 |  |
|  | +β2 | before | -26.0 ± 0.5 | 5.3 ± 0.3 | -41.0 ± 0.7 | 8.92 ± 0.5 | 0.33 (0.26, 0.39) |
|  |  | after | -23.3 ± 1.0 | 7.09 ± 0.5 | -43.1 ± 1.0 | 9.9 ± 0.8 |  |
| C918S | -β2 | before | -21.2 ± 0.5 | 5.8 ± 0.3 | -36.8 ± 0.9 | 7.8 ± 0.6 | 0.17 (0.12, 0.22) |
|  |  | after | >50 | 24.6 ± 6.5 | -56.8 ± 12.9 | 16.1 ± 9.8 |  |
|  | +β2 | before | -21.3 ± 0.3 | 5.3 ± 0.1 | -41.1 ± 1.0 | 6.7 ± 0.6 | 0.54 (0.43, 0.65) |
|  |  | after | -17.2 ± 0.7 | 7.2 ± 0.3 | -42.6 ± 0.8 | 8.7 ± 0.6 |  |
| C910S  C912S | -β2 | before | -22.6 ± 0.3 | 5.8 ± 0.1 | -45.1 ± 0.8 | 9.1 ± 0.4 | 0.14 (0.10, 0.18) |
|  |  | after | -9.2 ± 4.4 | 11.0 ± 1.0 | -53.0 ± 1.9 | 13.1 ± 1.3 |  |
|  | +β2 | before | -21.7 ± 0.3 | 6.1 ± 0.2 | -42.1 ± 0.5 | 9.9 ± 0.5 | 0.32 (0.20, 0.44) |
|  |  | after | -14.2 ± 3.3 | 10.6 ± 1.1 | -47.2 ± 1.0 | 13.0 ± 0.7 |  |
| C910S  C918S | -β2 | before | -15.7 ± 0.7 | 6.1 ± 0.4 | -36.1 ± 0.7 | 10.2 ± 0.7 | 0.24 (0.11, 0.37) |
|  |  | after | >50 | 19.5 ± 7.0 | -38.2 ± 3.5 | 8.0 ± 2.9 |  |
|  | +β2 | before | -18.8 ± 0.3 | 6.3 ± 0.2 | -43.0 ± 0.4 | 8.3 ± 0.3 | 0.23 (0.11, 0.36) |
|  |  | after | -0.23 ± 7.7 | 14.7 ± 1.6 | -47.6 ± 2.6 | 17.7 ± 2.8 |  |
| C912S  C918S | -β2 | before | -23.5 ± 0.4 | 5.6 ± 0.2 | -43.7 ± 0.8 | 7.1 ± 0.7 | 0.23 (0.15, 0.31) |
|  |  | after | -18.8 ± 2.5 | 9.4 ± 0.8 | -48.0 ± 2.0 | 10.3 ± 1.5 |  |
|  | +β2 | before | -22.8 ± 0.4 | 5.3 ± 0.3 | -41.9 ± 0.8 | 6.6 ± 0.7 | 0.68 (0.60, 0.76) |
|  |  | after | -17.7 ± 1.9 | 8.1 ± 0.8 | -45.1 ± 0.5 | 8.3 ± 0.5 |  |

**Table 3. Table providing values for fits of the data presented in Fig. 4 and Supplementary File 4.** G-V and SSI relationship data were fitted by a Boltzmann curve. V_1/2_ provides the midpoint voltage of the calculated curve (in mV) and Vc the unit-less slope, with standard error of the mean (SEM). Right column shows peak conductance after toxin treatment as a fraction of untreated peak conductance with the upper and lower bounds of the 95% confidence interval in parentheses, reflecting the data displayed in the dot plots.
