# Supplementary material for: Binary architecture of the Nav1.2-β2 signaling complex
Source: eLife. 2016 Feb 19;5:e10960. doi: 10.7554/eLife.10960 (PMC4769172; doi:10.7554/eLife.10960)
Supplement: Figure 4—source data 2. — G-V and SSI relationship data were fitted by a Boltzmann curve. 1/2 provides the midpoint voltage of the calculated curve (in mV) and Vc the unit-less slope, with standard error of the mean (SEM). Right column shows peak conductance after toxin treatment as a fraction of untreated peak conductance with the upper and lower bounds of the 95% confidence interval in parentheses, reflecting the data displayed in the dot plots. DOI: http://dx.doi.org/10.7554/eLife.10960.013 [file elife-10960-fig4-data2.docx]

|  | | | activation | | inactivation | | peak Gafter/peak Gbefore |
| --- | --- | --- | --- | --- | --- | --- | --- |
|  |  |  | V1/2 | Vc | V1/2 | Vc |  |
| hNav1.2 WT | -β2 | before | -30.9 ± 1.0 | 4.5 ± 0.3 | -41.1 ± 1.7 | 6.7 ± 0.7 | 0.59 (0.48, 0.71) |
|  |  | after | -24.1 ± 0.6 | 5.6 ± 0.2 | -44.4 ± 1.1 | 8.7 ± 0.6 |  |
|  |  | recovery | -31.2 ± 1.7 | 3.8 ± 0.4 | -45.2 ± 1.0 | 8.7 ± 0.5 | 0.80 (0.61, 0.99) |
|  | +β2 | before | -27.5 ± 0.5 | 4.3 ± 0.2 | -44.1 ± 1.1 | 9.1 ± 0.7 | 0.95 (0.87, 1.02) |
|  |  | after | -25.2 ± 0.2 | 4.5 ± 0.1 | -43.9 ± 1.2 | 9.6 ± 0.9 |  |
|  |  | recovery | -25.3 ± 0.6 | 4.6 ± 0.2 | -45.5 ± 1.1 | 10.4 ± 0.7 | 1.06 (0.86, 1.27) |

**Table 4. Table providing values for fits of the data presented in Supplementary File 5.** G-V and SSI relationship data were fitted by a Boltzmann curve. V_1/2_ provides the midpoint voltage of the calculated curve (in mV) and Vc the unit-less slope, with standard error of the mean (SEM). Right column shows peak conductance after toxin treatment as a fraction of untreated peak conductance with the upper and lower bounds of the 95% confidence interval in parentheses, reflecting the data displayed in the dot plots.
