# Supplementary material for: Binary architecture of the Nav1.2-β2 signaling complex
Source: eLife. 2016 Feb 19;5:e10960. doi: 10.7554/eLife.10960 (PMC4769172; doi:10.7554/eLife.10960)
Supplement: Figure 6—source data 1. — G-V and SSI relationship data were fitted by a Boltzmann curve. V1/2 provides the midpoint voltage of the calculated curve (in mV) and Vc the unit-less slope, with standard error of the mean (SEM). Right column shows peak conductance after toxin treatment as a fraction of untreated peak conductance with the upper and lower bounds of the 95% confidence interval in parentheses, reflecting the data displayed in the dot plots. DOI: http://dx.doi.org/10.7554/eLife.10960.019 [file elife-10960-fig6-data1.docx]

|  | | | activation | | inactivation | | peak Gafter/peak Gbefore |
| --- | --- | --- | --- | --- | --- | --- | --- |
|  |  |  | V1/2 | Vc | V1/2 | Vc |  |
| rNav1.2a WT | -β4 | before | -21.2 ± 0.2 | 6.4 ± 0.2 | -39.9 ± 0.6 | 9.8 ± 0.6 | 0.22 (0.08, 0.36) |
|  |  | after | -12.8 ± 0.8 | 9.4 ± 0.5 | -42.4 ± 1.7 | 11.6 ± 1.7 |  |
|  | +β4 | before | -24.3 ± 0.4 | 7.3 ± 0.3 | -47.6 ± 0.6 | 10.7 ± 0.6 | 0.55 (0.49, 0.61) |
|  |  | after | -21.8 ± 0.3 | 8.2 ± 0.2 | -53.2 ± 0.6 | 12.1 ± 0.5 |  |
| C910S | -β4 | before | -23.2 ± 0.3 | 6.6 ± 0.3 | -44.6 ± 0.8 | 12.1 ± 0.9 | 0.17 (0.02, 0.32) |
|  |  | after | -15.5 ± 0.6 | 9.9 ± 0.4 | -47.5 ± 1.4 | 14.6 ± 1.6 |  |
|  | +β4 | before | -20.9 ± 0.4 | 6.5 ± 0.4 | -47.1 ± 0.7 | 11.4 ± 0.7 | 0.11 (0.05, 0.17) |
|  |  | after | -7.9 ± 1.1 | 10.4 ± 0.5 | -49.9 ± 1.0 | 13.6 ± 1.0 |  |

**Table 5. Table providing values for fits of the data presented in Fig. 6.** G-V and SSI relationship data were fitted by a Boltzmann curve. V_1/2_ provides the midpoint voltage of the calculated curve (in mV) and Vc the unit-less slope, with standard error of the mean (SEM). Right column shows peak conductance after toxin treatment as a fraction of untreated peak conductance with the upper and lower bounds of the 95% confidence interval in parentheses, reflecting the data displayed in the dot plots.
