# Supplementary material for: Binary architecture of the Nav1.2-β2 signaling complex
Source: eLife. 2016 Feb 19;5:e10960. doi: 10.7554/eLife.10960 (PMC4769172; doi:10.7554/eLife.10960)
Supplement: Figure 7—source data 1. — G-V and SSI relationship data were fitted by a Boltzmann curve. V1/2 provides the midpoint voltage of the calculated curve (in mV) and Vc the unit-less slope, with standard error of the mean (SEM). Right column shows peak conductance after toxin treatment as a fraction of untreated peak conductance with the upper and lower bounds of the 95% confidence interval in parentheses, reflecting the data displayed in the dot plots. DOI: http://dx.doi.org/10.7554/eLife.10960.021 [file elife-10960-fig7-data1.docx]

|  | | | activation | | inactivation | | peak Gafter/peak Gbefore |
| --- | --- | --- | --- | --- | --- | --- | --- |
|  |  |  | V1/2 | Vc | V1/2 | Vc |  |
| hNav1.5 WT | -β2 | before | -35.6 ± 0.4 | 5.9 ± 0.2 | -67.8 ± 0.7 | 7.7 ± 0.4 | 0.29 (0.22, 0.37) |
|  |  | after | -31.1 ± 0.8 | 7.3 ± 0.4 | -65.0 ± 1.3 | 6.8 ± 0.8 |  |
|  | +β2 | before | -35.2 ± 0.5 | 5.9 ± 0.2 | -70.3 ± 0.7 | 7.6 ± 0.4 | 0.28 (0.15, 0.39) |
|  |  | after | -28.5 ± 1.1 | 7.9 ± 0.5 | -71.3 ± 1.4 | 8. 3 ± 0.8 |  |

**Table 6. Table providing values for fits of the data presented in Fig. 7.** G-V and SSI relationship data were fitted by a Boltzmann curve. V_1/2_ provides the midpoint voltage of the calculated curve (in mV) and Vc the unit-less slope, with standard error of the mean (SEM). Right column shows peak conductance after toxin treatment as a fraction of untreated peak conductance with the upper and lower bounds of the 95% confidence interval in parentheses, reflecting the data displayed in the dot plots.
