# Supplementary material for: Binary architecture of the Nav1.2-β2 signaling complex
Source: eLife. 2016 Feb 19;5:e10960. doi: 10.7554/eLife.10960 (PMC4769172; doi:10.7554/eLife.10960)
Supplement: Figure 8—source data 1. — G-V and SSI relationship data were fitted by a Boltzmann curve. V1/2 provides the midpoint voltage of the calculated curve (in mV) and Vc the unit-less slope, with standard error of the mean (SEM). Right column shows peak conductance after toxin treatment as a fraction of untreated peak conductance with the upper and lower bounds of the 95% confidence interval in parentheses, reflecting the data displayed in the dot plots. DOI: http://dx.doi.org/10.7554/eLife.10960.023 [file elife-10960-fig8-data1.docx]

|  | | | activation | | inactivation | | peak Gafter/peak Gbefore |
| --- | --- | --- | --- | --- | --- | --- | --- |
|  |  |  | V1/2 | Vc | V1/2 | Vc |  |
| AahII | -β2 | before | -16.1 ± 0.2 | 5.5 ± 0.1 | -36.5 ± 0.7 | 7.1 ± 0.6 | 1.74 (1.61, 1.88) |
|  |  | after | -24.7 ± 0.6 | 6.4 ± 0.2 | -52.0 ± 1.2 | 6.7 ± 0.9 |  |
|  | +β2 | before | -11.8 ± 0.3 | 5.8 ± 0.1 | -33.8 ± 0.3 | 9.7 ± 0.3 | 1.68 (1.44, 1.93) |
|  |  | after | -23.1 ± 0.6 | 5.8 ± 0.2 | -46.7 ± 1.3 | 5.8 ± 1.0 |  |
| PaurTx3 | -β2 | before | -16.0 ± 0.3 | 5.7 ± 0.1 | -37.1 ± 0.7 | 7.3 ± 0.7 | 0.63 (0.47, 0.78) |
|  |  | after | -15.7 ± 1.7 | 6.2 ± 0.5 | -38.6 ± 0.5 | 8.3 ± 0.4 |  |
|  | +β2 | before | -12.2 ± 0.4 | 6.1 ± 0.2 | -32.9 ± 0.6 | 8.8 ± 0.6 | 0.65 (0.60, 0.70) |
|  |  | after | -7.3 ± 2.9 | 8.2 ± 0.7 | -34.7 ± 0.8 | 11.5 ± 0.7 |  |
| ProTx-I | -β2 | before | -16.6 ± 0.4 | 5.0 ± 0.1 | -38.1 ± 0.7 | 7.4 ± 0.6 | 0.17 (0.09, 0.26) |
|  |  | after | -9.2 ± 3.0 | 7.8 ± 0.7 | -42.2 ± 1.1 | 9.0 ± 0.6 |  |
|  | +β2 | before | -15.6 ± 0.6 | 5.1 ± 0.2 | -34.2 ± 0.7 | 8.5 ± 0.5 | 0.23 (0.17, 0.30) |
|  |  | after | -0.6 ± 7.2 | 9.3 ± 1.2 | -39.2 ± 1.1 | 10.7 ± 0.9 |  |
| ProTx-II | -β2 | before | -17.3 ± 0.5 | 5.4 ± 0.2 | -39.3 ± 1.0 | 7.1 ± 0.6 | 0.31 (0.20, 0.42) |
|  |  | after | -9.3 ± 2.5 | 8.5 ± 0.7 | -42.2 ± 0.7 | 9.6 ± 0.6 |  |
|  | +β2 | before | -21.4 ± 0.3 | 5.5 ± 0.2 | -37.8 ± 1.1 | 6.8 ± 0.9 | 0.77 (0.64, 0.90) |
|  |  | after | -18.9 ± 0.6 | 6.7 ± 0.3 | -42.0 ± 1.0 | 8.8 ± 0.6 |  |

**Table 7. Table providing values for fits of the data presented in Fig. 8 and Supplementary File 6.** G-V and SSI relationship data were fitted by a Boltzmann curve. V_1/2_ provides the midpoint voltage of the calculated curve (in mV) and Vc the unit-less slope, with standard error of the mean (SEM). Right column shows peak conductance after toxin treatment as a fraction of untreated peak conductance with the upper and lower bounds of the 95% confidence interval in parentheses, reflecting the data displayed in the dot plots.
